# A New Hybrid Particle Swarm Optimization–Teaching–Learning-Based Optimization for Solving Optimization Problems

**DOI:** 10.3390/biomimetics9010008

**Published:** 2023-12-25

**Authors:** Štěpán Hubálovský, Marie Hubálovská, Ivana Matoušová

**Affiliations:** 1 Department of Applied Cybernetics, Faculty of Science, University of Hradec Králové, 50003 Hradec Kralove, Czech Republic; 2 Department of Technics, Faculty of Education, University of Hradec Králové, 50003 Hradec Kralove, Czech Republic; marie.hubalovska@uhk.cz; 3 Department of Mathematics, Faculty of Science, University of Hradec Králové, 50003 Hradec Kralove, Czech Republic; ivana.matousova@uhk.cz

**Keywords:** optimization, metaheuristic, particle swarm optimization, teaching–learning-based optimization, hybrid-based algorithm, exploration, exploitation

## Abstract

This research paper develops a novel hybrid approach, called hybrid particle swarm optimization–teaching–learning-based optimization (hPSO-TLBO), by combining two metaheuristic algorithms to solve optimization problems. The main idea in hPSO-TLBO design is to integrate the exploitation ability of PSO with the exploration ability of TLBO. The meaning of “exploitation capabilities of PSO” is the ability of PSO to manage local search with the aim of obtaining possible better solutions near the obtained solutions and promising areas of the problem-solving space. Also, “exploration abilities of TLBO” means the ability of TLBO to manage the global search with the aim of preventing the algorithm from getting stuck in inappropriate local optima. hPSO-TLBO design methodology is such that in the first step, the teacher phase in TLBO is combined with the speed equation in PSO. Then, in the second step, the learning phase of TLBO is improved based on each student learning from a selected better student that has a better value for the objective function against the corresponding student. The algorithm is presented in detail, accompanied by a comprehensive mathematical model. A group of benchmarks is used to evaluate the effectiveness of hPSO-TLBO, covering various types such as unimodal, high-dimensional multimodal, and fixed-dimensional multimodal. In addition, CEC 2017 benchmark problems are also utilized for evaluation purposes. The optimization results clearly demonstrate that hPSO-TLBO performs remarkably well in addressing the benchmark functions. It exhibits a remarkable ability to explore and exploit the search space while maintaining a balanced approach throughout the optimization process. Furthermore, a comparative analysis is conducted to evaluate the performance of hPSO-TLBO against twelve widely recognized metaheuristic algorithms. The evaluation of the experimental findings illustrates that hPSO-TLBO consistently outperforms the competing algorithms across various benchmark functions, showcasing its superior performance. The successful deployment of hPSO-TLBO in addressing four engineering challenges highlights its effectiveness in tackling real-world applications.

## 1. Introduction

Optimization is the process of finding the best solution among all available solutions for an optimization problem [1]. From a mathematical point of view, every optimization problem consists of three main parts: decision variables, constraints, and objective function. Therefore, the goal in optimization is to determine the appropriate values for the decision variables so that the objective function is optimized by respecting the constraints of the problem [2]. There are countless optimization problems in science, engineering, industry, and real-world applications that must be solved using appropriate techniques [3].

Metaheuristic algorithms are one of the most effective approaches used in handling optimization tasks. Metaheuristic algorithms are able to provide suitable solutions for optimization problems without the need for gradient information, only based on random search in the problem solving space, using random operators and trial and error processes [4]. Advantages such as simple concepts, easy implementation, efficiency in nonlinear, nonconvex, discontinuous, nonderivative, NP-hard optimization problems, and efficiency in discrete and unknown search spaces have led to the popularity of metaheuristic algorithms among researchers [5]. The optimization process in metaheuristic algorithms starts with the random generation of a number of solvable solutions for the problem. Then, during an iteration-based process, these initial solutions are improved based on algorithm update steps. At the end, the best improved solution is presented as the solution to the problem [6]. The nature of random search in metaheuristic algorithms means that there is no guarantee of achieving the global optimum using these approaches. However, due to the proximity of the solutions provided by metaheuristic algorithms to the global optimum, they are acceptable as quasi-optimal solutions [7].

In order to perform the search process in the problem-solving space well, metaheuristic algorithms must be able to scan the problem-solving space well at both global and local levels. Global search with the concept of exploration leads to the ability of the algorithm to search all the variables in the search space in order to prevent the algorithm from getting stuck in the local optimal areas and to accurately identify the main optimal area. Local search with the concept of exploitation leads to the ability of the algorithm to search accurately and meticulously around the discovered solutions and promising areas with the aim of achieving solutions that are close to the global optimum. In addition to the ability in exploration and exploitation, what leads to the success of the metaheuristic algorithm in providing a suitable search process is its ability to establish a balance between exploration and exploitation during the search process [8]. The desire of researchers to obtain better solutions for optimization problems has led to the design of numerous metaheuristic algorithms.

The main question of this research whether, considering the many metaheuristic algorithms that have been introduced so far, there is a need to design newer algorithms or develop hybrid approaches from the combination of several metaheuristic algorithms. In response to this question, the no free lunch (NFL) [9] theorem explains that no unique metaheuristic algorithm is the best optimizer for all optimization applications. According to the NFL theorem, the proper performance of a metaheuristic algorithm in solving a set of optimization problems is not a guarantee of the same performance of that algorithm in handling other optimization applications. Therefore, the NFL theorem, by keeping the research field active, motivates researchers to be able to provide more effective solutions for optimization problems by introducing new algorithms as well as developing hybrid versions of the combination of several algorithms.

Numerous metaheuristic algorithms have been designed by researchers. Among these, particle swarm optimization (PSO) [10] and teaching–learning-based optimization (TLBO) [11] are successful and popular algorithms that have been widely employed to deal with optimization problems in various sciences.

The design of PSO is inspired by the movement of flocks of birds and fish in search of food. In PSO design, the position of the best member is used to update the position of the population members. This dependence of the update process on the best member prevents the algorithm from scanning the entire problem-solving space, and as a result, it can lead to the rapid convergence of the algorithm in inappropriate local optima. Therefore, improving the exploration ability in PSO in order to manage the global search plays a significant role in the more successful performance of this algorithm.

In the design of TLBO, it is adapted from the exchange of knowledge between the teacher and students and the students with each other in the educational space of the classroom. The teacher phase in the design of TLBO is such that it has led to the high capability of this algorithm in exploration and global search.

The innovation and novelty of this article are in developing a new hybrid metaheuristic algorithm called hybrid particle swarm optimization–teaching–learning-based optimization (hPSO-TLBO), which is used in handling optimization tasks. The main motivation in designing hybrid algorithms is to benefit from the advantages of two or more algorithms at the same time by combining them. PSO has good quality in exploitation, but on the other hand, it suffers from the weakness of exploration. On the other hand, TLBO has high quality in exploration. Therefore, the main goal in designing hPSO-TLBO is to design a powerful hybrid metaheuristic approach with benefit and combination the exploitation power of PSO and the exploration power of TLBO.

The main contributions of this paper are as follows:

- hPSO-TLBO is developed based on the combination of particle swarm optimization–teaching–learning-based optimization.
- The performance of hPSO-TLBO is tested on fifty-two standard benchmark functions from unimodal, high-dimensional multimodal, fixed-dimensional multimodal types, and the CEC 2017 test suite.
- The performance of hPSO-TLBO is evaluated in handling real-world applications, challenged on four design engineering problems.
- The results of hPSO-TLBO are compared with the performance of twelve well-known metaheuristic algorithms.

This paper is organized as follows: the literature review is presented in Section 2. The proposed hPSO-TLBO approach is introduced and modeled in Section 3. Simulation studies and results are presented in Section 4. The effectiveness of hPSO-TLBO in handling real-world applications is challenged in Section 5. Finally, conclusions and suggestions for future research are provided in Section 6.

## 2. Literature Review

Various natural phenomena have inspired metaheuristic algorithms, the behavior of living organisms in nature, genetics, and biology, laws and concepts of physics, rules of games, human behavior, and other evolutionary phenomena. Based on the source of inspiration in the design, metaheuristic algorithms are placed in five groups: swarm-based, evolutionary-based, physics-based, game-based, and human-based.

Swarm-based metaheuristic algorithms have been proposed based on modeling swarm behaviors among birds, animals, insects, aquatic animals, plants, and other living organisms in nature. The most famous algorithms of this group are particle swarm optimization (PSO) [10], artificial bee colony (ABC) [12], ant colony optimization (ACO) [13], and firefly algorithm (FA) [14]. The PSO algorithm was developed using inspiration from the movement of flocks of birds and fishes searching for food. ABC was proposed based on the activities of honey bees in a colony, aiming to access food resources. ACO was introduced based on modeling the ability of ants to discover the shortest path between the colony and the food source. FA was developed using inspiration from optical communication between fireflies. Foraging, hunting, migration, digging are among the most common natural behaviors among living organisms, which have been a source of inspiration in the design of swarm-based metaheuristic algorithms such as the coati optimization algorithm (COA) [15], whale optimization algorithm (WOA) [16], white shark optimizer (WSO) [17], reptile search algorithm (RSA) [18], pelican optimization algorithm (POA) [19], kookaburra optimization algorithm (KOA) [20], grey wolf optimizer (GWO) [21], walruses optimization algorithm (WaOA) [22], golden jackal optimization (GJO) [23], honey badger algorithm (HBA) [24], lyrebird optimization algorithm (LOA) [25], marine predator algorithm (MPA) [26], African vultures optimization algorithm (AVOA) [27], and tunicate swarm algorithm (TSA) [28].

Evolutionary-based metaheuristic algorithms have been proposed based on modeling concepts of biology and genetics such as survival of the fittest, natural selection, etc. The genetic algorithm (GA) [29] and differential evolution (DE) [30] are among the most well-known and widely used metaheuristic algorithms developed based on the modeling of the generation process, Darwin’s evolutionary theory, and the use of mutation, crossover, and selection random evolutionary operators. Artificial immune system (AIS) [31] algorithms are designed with inspiration from the human body’s defense mechanism against diseases and microbes.

Physics-based metaheuristic algorithms have been proposed based on modeling concepts, transformations, forces, laws in physics. Simulated annealing (SA) [32] is one of the most famous metaheuristic algorithms of this group, which was developed based on the modeling of the annealing process of metals, during which, based on physical transformations, metals are melted under heat and then slowly cooled to become the crystal of its idea. Physical forces have inspired the design of several algorithms, including the gravitational search algorithm (GSA) [33], based on gravitational force simulation; spring search algorithm (SSA) [34], based on spring potential force simulation; and momentum search algorithm (MSA) [35], based on impulse force simulation. Some of the most popular physics-based methods are water cycle algorithm (WCA) design [36], electromagnetism optimization (EMO) [37], the Archimedes optimization algorithm (AOA) [38], Lichtenberg algorithm (LA) [39], equilibrium optimizer (EO) [40], black hole algorithm (BHA) [41], multi-verse optimizer (MVO) [42], and thermal exchange optimization (TEO) [43].

Game-based metaheuristic algorithms have been proposed, inspired by governing rules, strategies of players, referees, coaches, and other influential factors in individual and group games. The modeling of league matches was a source of inspiration in designing algorithms such as football game-based optimization (FGBO) [44], based on a football game, and the volleyball premier league (VPL) algorithm [45], based on a volleyball league. The effort of players in a tug-of-war competition was the main idea in the design of tug of war optimization (TWO) [46]. Some other game-based algorithms are the golf optimization algorithm (GOA) [47], hide object game optimizer (HOGO) [48], darts game optimizer (DGO) [49], archery algorithm (AA) [5], and puzzle optimization algorithm (POA) [50].

Human-based metaheuristic algorithms have been proposed, inspired by strategies, choices, decisions, thoughts, and other human behaviors in individual and social life. Teaching–learning-based optimization (TLBO) [11] is one of the most famous human-based algorithms, which is designed based on modeling the classroom learning environment and the interactions between students and teachers. Interactions between doctors and patients in order to treat patients is the main idea in the design of doctor and patient optimization (DPO) [51]. Cooperation among the people of a team in order to achieve the set goals of that team is employed in teamwork optimization algorithm (TOA) [52] design. The efforts of both the poor and the rich sections of the society in order to improve their economic situation were a source of inspiration in the design of poor and rich optimization (PRO) [53]. Some of the other human-based metaheuristic algorithms are the mother optimization algorithm (MOA) [54], herd immunity optimizer (CHIO) [55], driving training-based optimization (DTBO) [56], Ali Baba and the Forty Thieves (AFT) [57], election-based optimization algorithm (EBOA) [58], chef-based optimization algorithm (ChBOA) [59], sewing training-based optimization (STBO) [60], language education optimization (LEO) [61], gaining–sharing knowledge-based algorithm (GSK) [62], and war strategy optimization (WSO) [63].

In addition to the groupings stated above, researchers have developed hybrid metaheuristic algorithms by combining two or more metaheuristic algorithms. The main goal and motivation in the construction of hybrid metaheuristic algorithms is to take advantage of several algorithms at the same time in order to improve the performance of the optimization process compared to the single versions of each of the combined algorithms. The combination of TLBO and HS was used to design the hTLBO-HS hybrid approach [64]. hPSO-YUKI was proposed based on the combination of PSO and the YUKI algorithm to address the challenge of double crack identification in CFRP cantilever beams [65]. The hGWO-PSO hybrid approach was designed by integrating GWO and PSO for static and dynamic crack identification [66].

PSO and TLBO algorithms are successful metaheuristic approaches that have always attracted the attention of researchers and have been employed to solve many optimization applications. In addition to using single versions of PSO and TLBO, researchers have tried to develop hybrid approaches by integrating these two algorithms that benefit from the advantages of both algorithms at the same time. A hybrid version of hPSO-TLBO was proposed based on merging the better half of the PSO population and the better half obtained from the TLBO teacher phase. Then, the merged population enters the learner phase of TLBO. In this hybrid approach, there is no change or integration in the equations [67]. A hybrid version of hPSO-TLBO based on population merging was proposed for trajectory optimization [68]. The idea of dividing and merging the population has also been used to solve optimization problems [69]. A hybrid version of PSO and TLBO was proposed for distribution network reconfiguration [70]. A hybrid version of TLBO and SA as well as the use of a support vector machine was developed for gene expression data [71]. From the combination of the sine–cosine algorithm and TLBO, the hSCA-TLBO hybrid approach was proposed for visual tracking [72]. Sunflower optimization and TLBO were combined to develop hSFO-TLBO for biodegradable classification [73]. A hybrid version called hTLBO-SSA was proposed from the combination of the salp swarm algorithm and TLBO for reliability redundancy allocation problems [74]. A hybrid version consisting of PSO and SA was developed under the title of hPSO-SA for mobile robot path planning in warehouses [75]. Harris hawks optimization and PSO were integrated with Ham to design hPSO-HHO for renewable energy applications [76]. A hybrid version called hPSO-GSA was proposed from the combination of PSO and GSA for feature selection [77]. A hybrid version made from PSO and GWO called hPSO-GWO was developed to deal with reliability optimization and redundancy allocation for fire extinguisher drones [78]. A hybrid PSO-GA approach was proposed for flexible flow shop scheduling with transportation [79].

In addition to the development of hybrid metaheuristic algorithms, researchers have tried to improve existing versions of algorithms by making modifications. Therefore, numerous improved versions of metaheuristic algorithms have been proposed by scientists to improve the performance of the original versions of existing algorithms. An improved version of PSO was proposed for efficient maximum power point tracking under partial shading conditions [80]. An improved version of PSO was developed based on hummingbird flight patterns to enhance search quality and population diversity [81]. In order to deal with the planar graph coloring problem, an improved version of PSO was designed [82]. The application of an improved version of PSO was evaluated for the optimization of reactive power [83]. An improved version of TLBO for optimal placement and sizing of electric vehicle charging infrastructure in a grid-tied DC microgrid was proposed [84]. An improved version of TLBO was developed for solving time–cost optimization in generalized construction projects [85]. Two improved TLBO approaches were developed for the solution of inverse boundary design problems [86]. In order to address the challenge of selective harmonic elimination in multilevel inverters, an improved version of TLBO was designed [87].

Based on the best knowledge from the literature review, although several attempts have been made to improve the performance of PSO and TLBO algorithms and also to design hybrid versions of these two algorithms, it is still possible to develop an effective hybrid approach to solve optimization problems by integrating the equations of these two algorithms and making modifications in their design. In order to address this research gap in the study of metaheuristic algorithms, in this paper, a new hybrid metaheuristic approach combining PSO and TLBO was developed, which is discussed in detail in the next section.

## 3. Hybrid Particle Swarm Optimization–Teaching–Learning-Based Optimization

In this section, PSO and TLBO are discussed first, and their mathematical equations are presented. Then, the proposed hybrid particle swarm optimization–teaching–learning-based optimization (hPSO-TLBO) approach is presented based on the combination of PSO and TLBO.

### 3.1. Particle Swarm Optimization (PSO)

PSO is a prominent swarm-based metaheuristic algorithm widely known for its ability to emulate the foraging behavior observed in fish and bird flocks, enabling an effective search for optimal solutions. All PSO members are candidate solutions representing values of decision variables based on their position in the search space. The personal best experience $P_{best_{i}}$ and the collective best experience $g_{best}$ are used in PSO design in the population updating process. $P_{best_{i}}$ represents the best candidate solution that each PSO member has been able to achieve up to the current iteration. $g_{best}$ is the best candidate solution discovered up to the current iteration by the entire population in the search space. The population update equations in PSO are as follows:

(1) $$X_{i}(t+1)=X_{i}(t)+V_{i}(t),$$

(2) $$V_{i}(t+1)=\omega \left(t\right)\cdot V_{i}(t)+r_{1}{\cdot c}_{1}\cdot \left(P_{best_{i}}-X_{i}(t)\right)+r_{2}{\cdot c}_{2}\cdot \left(g_{best}-X_{i}(t)\right),$$

(3) $$\omega (t)=0.9-0.8\cdot \frac{t-1}{T-1}$$

where $X_{i}(t)$ is the ith PSO member, $V_{i}(t)$ is its velocity, $P_{best_{i}}$ is the best obtained solution so far by the ith PSO member, $g_{best}$ is the best obtained solution so far by overall PSO population, ω(t) is the inertia weight factor with linear reduction from 0.9 to 0.1 during algorithm iteration, T is the maximum number of iterations, t is the iteration counter, $r_{1}$ and $r_{2}$ are the real numbers with a uniform probability distribution between 0 and 1 (i.e., $r_{1},r_{2}\in U[0,1]$), $c_{1}$ and $c_{2}$ (fulfilling the condition $c_{1}+c_{2}\leq 4$) are acceleration constants in which $c_{1}$ represents the confidence of a PSO member in itself while $c_{2}$ represents the confidence of a PSO member in the population.

### 3.2. Teaching–Learning-Based Optimization (TLBO)

TLBO has established itself as a leading and extensively employed human-based metaheuristic algorithm, effectively simulating the dynamics of educational interactions within a classroom setting. Like PSO, each TLBO member is also a candidate solution to the problem based on its position in the search space. In the design of TLBO, the best member of the population with the most knowledge is considered a teacher, and the other population members are considered class students. In TLBO, the position of population members is updated under two phases (the teacher and learner phases).

In the teacher phase, the best member of the population with the highest level of knowledge, denoted as the teacher, tries to raise the academic level of the class by teaching and transferring knowledge to students. The population update equations in TLBO based on the teacher phase are as follows:

(4) $$X_{i}=X_{i}+r_{3}\cdot (T-I\cdot M),$$

(5) $$M=\frac{\sum_{i=1}^{N}X_{i}}{N},$$

where $X_{i}$ is the ith TLBO member, T is the teacher, M is the mean value of the class, $r_{3}\in U[0,1]$, I is a random integer obtained from a uniform distribution on the set $\left\{1,2\right\}$, and N represents the number of population members.

In the learner phase, the students of the class try to improve their knowledge level and thus the class by helping each other. In TLBO, it is assumed that each student randomly chooses another student and exchanges knowledge. The population update equations in TLBO based on the learner phase are as follows:

(6) $$X_{i}=\left\{\begin{array}{l} X_{i}+r_{4}\cdot (X_{k}-X_{i}),\;F_{k}<F_{i};\\ X_{i}+r_{4}\cdot (X_{i}-X_{k}),\;else, \end{array}\right.$$

where $X_{k}$ is the kth student ($k\in \left\{1,2,3,\;...,\;N\right\}\;and\;k\neq i$), $F_{k}$ is its objective function value, and $r_{4}\in U[0,1]$.

### 3.3. Proposed Hybrid Particle Swarm Optimization–Teaching–Learning-Based Optimization (hPSO-TLBO)

This subsection presents the introduction and modeling of the proposed hPSO-TLBO approach, which combines the features of PSO and TLBO. In this design, an attempt was made to use the advantages of each of the mentioned algorithms so as to develop a hybrid metaheuristic algorithm that performs better than PSO or TLBO.

PSO has a high exploitation ability based on the term $r_{1}\cdot c_{1}\cdot \left(P_{best_{i}}-X_{i}\right)$ in the update equations; however, due to the dependence of the update process on the best population member $g_{best}$, PSO is weak in global search and exploration. In fact, the term $r_{2}{\cdot c}_{2}\cdot \left(g_{best}-X_{i}\right)$ in PSO can stop the algorithm by taking it to the local optimum and reaching the stationary state (early gathering of all population members in a solution).

The teacher phase in TLBO incorporates large and sudden changes in the population’s position, based on term $r_{3}\cdot (T-I\cdot M)$, resulting in global search and exploration capabilities. Enhancing exploration in metaheuristic algorithms improves the search process, preventing it from getting trapped in local optima and accurately identifying the main optimal area. Hence, the primary concept behind the design of the proposed hPSO-TLBO approach is to facilitate the exploration phase in PSO by leveraging the exceptional global search and exploration capabilities of TLBO. According to this, in hPSO-TLBO, a new hybrid metaheuristic algorithm is designed by integrating the exploration ability of TLBO with the exploitation ability of PSO.

For the possibility and effectiveness of the combination of PSO and TLBO, the term $r_{2}\cdot c_{2}\cdot \left(g_{best}-X_{i}\right)$ was removed from Equation (2) (i.e., equation for velocity), and conversely, to improve the discovery ability, the term $r_{3}\cdot (T-I\cdot M)$ from the teacher phase of TLBO was added to this equation. Therefore, the new form of velocity equation in the hPSO-TLBO is as follows:

(7) $$V_{i}={\omega (t)\cdot V}_{i}+r_{1}\cdot c_{1}\cdot \left(P_{best_{i}}-X_{i}\right)+r_{3}\cdot (T-I\cdot M).$$

Then, based on the velocity calculated from Equation (7), and based on Equation (1), a new location for any hPSO-TLBO member is calculated by Equation (8). If the value of the objective function improves at the new location, it supersedes the previous position of the corresponding member based on Equation (9).

(8) $$X_{i}^{new}=X_{i}+V_{i},\;$$

(9) $$X_{i}=\left\{\begin{array}{c} X_{i}^{new},\;F_{i}^{new}\leq F_{i};\\ X_{i},\;else, \end{array}\right.$$

where $X_{i}^{new}$ is the new proposed location for the ith population member in the search space and $F_{i}^{new}$ is objective function value of $X_{i}^{new}$.

During the student phase of TLBO, every student chooses another student at random for the purpose of exchanging knowledge. A randomly selected student may have a better or worse knowledge status compared to the student who is the selector. In hPSO-TLBO design, an enhancement is introduced in the student phase, assuming that each student selects a superior student to elevate their knowledge level and enhance overall performance. In this case, if the objective function value of a member represents the scientific level of that member, the set of better students for each hPSO-TLBO member is determined using Equation (10):

(10) $$CS_{i}=\left\{X_{l};\;F_{k}<F_{l}\wedge l\in \left\{1,2,\;\ldots ,N\right\}\right\}\cup T,$$

where $CS_{i}$ is the set of suitable students for guiding the ith member $X_{i}$, and $X_{l}$ is the population member with a better objective function value $F_{l}$ than member $X_{i}$.

In the implementation of hPSO-TLBO, each student uniformly randomly chooses one of the higher-performing students from a given set and proceeds to exchange knowledge with them. Based on the exchange of knowledge in the student phase, a new location of each member is calculated by Equation (11). If the new position leads to an improvement in the objective function value, it replaces the previous position of the corresponding member, as specified by Equation (12).

(11) $$X_{i}^{new}=X_{i}+r_{4}(SS_{i}-X_{i}),\;$$

(12) $$X_{i}=\left\{\begin{array}{c} X_{i}^{new},\;F_{i}^{new}\leq F_{i};\\ X_{i},\;else, \end{array}\right.$$

where $SS_{i}$ is the selected student for guiding the ith population member.

**Figure 1:**
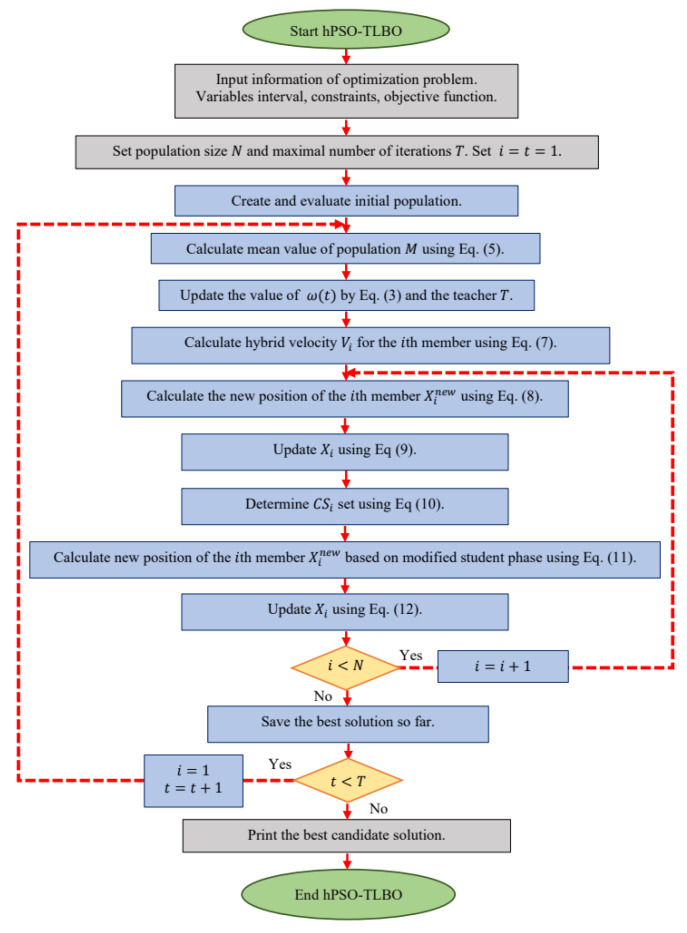
Flowchart of hPSO-TLBO.

Figure 1 presents a flowchart illustrating the implementation steps of the hPSO-TLBO approach, while Algorithm 1 provides the corresponding pseudocode.
**Algorithm 1.** Pseudocode of hPSO-TLBOStart hPSO-TLBO.1.Input problem information: variables, objective function, and constraints.2.Set the population size N and the maximum number of iterations T.3.Generate the initial population matrix at random.4.Evaluate the objective function.5.
For t=1 to T6.
Update the value of $\omega \left(t\right)$ by Equation (3) and the value of the teacher T.7.
Calculate M using Equation (5). $M\leftarrow \frac{\sum_{i=1}^{N}X_{i}}{N}$
8.
For i=1 to N
9.

Update $P_{best_{i}}$ based on comparison $X_{i}$ with $P_{best_{i}}$.10.

Set the best population member as teacher *T.*11.

Calculate hybrid velocity for the ith member using Equation (7). $V_{i}\leftarrow {\omega (t)\cdot V}_{i}+r_{1}\cdot c_{1}\cdot \left(P_{best_{i}}-X_{i}\right)+r_{3}\cdot (T-I\cdot M)$
12.

Calculate new position of the ith population member using Equation (8). $X_{i}^{new}\leftarrow X_{i}+V_{i}$
13.

Update the ith member using Equation (9). $X_{i}\leftarrow \left\{\begin{array}{c} X_{i}^{new},\;F_{i}^{new}\leq F_{i};\\ X_{i},\;else. \end{array}\right.$
14.

Determine candidate students set for the ith member using Equation (10). $CS_{i}\leftarrow \left\{X_{k}|\;F_{k}<F_{i},\;k\in \left\{1,2,\;\ldots ,N\right\}\right\}\cup T$
15.

Calculate the new position of the ith population member based on modified student phase by Equation (11). $X_{i}^{new}\leftarrow X_{i}+r_{4}\cdot (SS_{i}-X_{i})$
16.

Update the ith member using Equation (12). $X_{i}\leftarrow \left\{\begin{array}{c} X_{i}^{new},\;F_{i}^{new}\leq F_{i};\\ X_{i},\;else. \end{array}\right.$
17.
end18.

Save the best candidate solution so far.19.
end 20.  Output the best quasi-optimal solution obtained with hPSO-TLBO.End hPSO-TLBO.

### 3.4. Computational Complexity of hPSO-TLBO

This subsection focuses on evaluating the computational complexity of the hPSO-TLBO algorithm. The initialization of hPSO-TLBO for an optimization problem with m decision variables has a computational complexity of O(Nm), where N represents the number of population members. In each iteration, the position of the population members in the search space is updated in two steps. As a result, in each iteration, the value of the objective function for each population member is computed twice. Hence, the computational complexity of the population update process in hPSO-TLBO is $O\left(2NmT\right),$ with T representing the total number of the algorithm’s iterations. Based on these, the overall computational complexity of the proposed hPSO-TLBO approach is O(Nm(2T+1)).

Similarly, the computational complexity of each of the PSO and TLBO algorithms can also be evaluated. PSO has a computational complexity of O(Nm(T+1)) and TLBO has a computational complexity of O(Nm(2T+1)). Therefore, from the point of view of computational complexity, the proposed hPSO-TLBO approach has a similar situation to TLBO, but compared to PSO, it has twice the computational complexity. Actually, the number of function evaluations in each iteration in hPSO-TLBO and TLBO is equal to 2*N* and in PSO is equal to *N*.

## 4. Simulation Studies and Results

In this section, the performance of the proposed hPSO-TLBO approach in solving optimization problems is evaluated. For this purpose, a set of fifty-two standard benchmark functions of unimodal, high-dimensional multimodal, and fixed-dimensional multimodal types [88], and CEC 2017 test suite [89] were employed.

### 4.1. Performance Comparison and Experimental Settings

In order to check the quality of hPSO-TLBO, the obtained results were compared with the performance of twelve well-known metaheuristic algorithms: PSO, TLBO, improved PSO (IPSO) [81], improved TLBO (ITLBO) [87], hybrid PSO-TLBO (hPT1) developed in [67], hybrid PSO-TLBO (hPT2) developed in [90], GWO, MPA, TSA, RSA, AVOA, WSO. Therefore, hPSO-TLBO was compared with twelve metaheuristic algorithms in total. The experiments were carried out on a Windows 10 computer with a 2.2GHz Core i7 processor and 16 GB of RAM, utilizing MATLAB 2018a as the software environment. The optimization results are reported using six statistical indicators: mean, best, worst, standard deviation (std), median, and rank. In addition, the value of the mean index was used to rank the metaheuristic algorithms in handling each of the benchmark functions.

### 4.2. Evaluation of Unimodal Test Functions F1 to F7

Unimodal functions are valuable for evaluating the exploitation and local search capabilities of metaheuristic algorithms since they lack local optima. Table 1 presents the optimization results of unimodal functions F1 to F7, obtained using hPSO-TLBO and other competing algorithms. The optimization results demonstrate that hPSO-TLBO excels in local search and exploitation, consistently achieving the global optimum for functions F1 to F6. Furthermore, hPSO-TLBO emerged as the top-performing optimizer for solving function F7. The analysis of simulation outcomes confirms that hPSO-TLBO, with its exceptional exploitation capability and superior results, outperforms competing algorithms in tackling functions F1 to F7 of unimodal type.

### 4.3. Evaluation of High-Dimensional Multimodal Test Functions F8 to F13

Due to having multiple local optima, high-dimensional multimodal functions are suitable options for global exploration and search in metaheuristic algorithms. The results of implementing hPSO-TLBO and competing algorithms on high-dimensional multimodal benchmarks F8 to F13 are presented in Table 2. Based on the results, hPSO-TLBO, with high discovery ability, was able to handle functions F9 and F11 while identifying the main optimal area, converging to the global optimum. The hPSO-TLBO demonstrates exceptional performance as the top optimizer for benchmarks F8, F10, F12, and F13. The simulation results clearly indicate that hPSO-TLBO, with its remarkable exploration capability, outperforms competing algorithms in effectively handling benchmarks F8 to F13 of high-dimensional multimodal type.

### 4.4. Evaluation of Fixed-Dimensional Multimodal Test Functions F14 to F23

**Figure 2:**
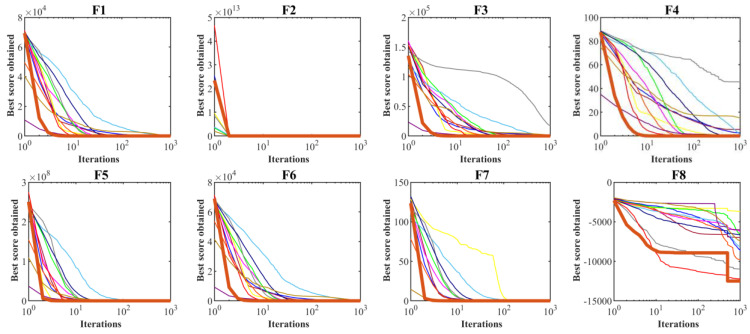

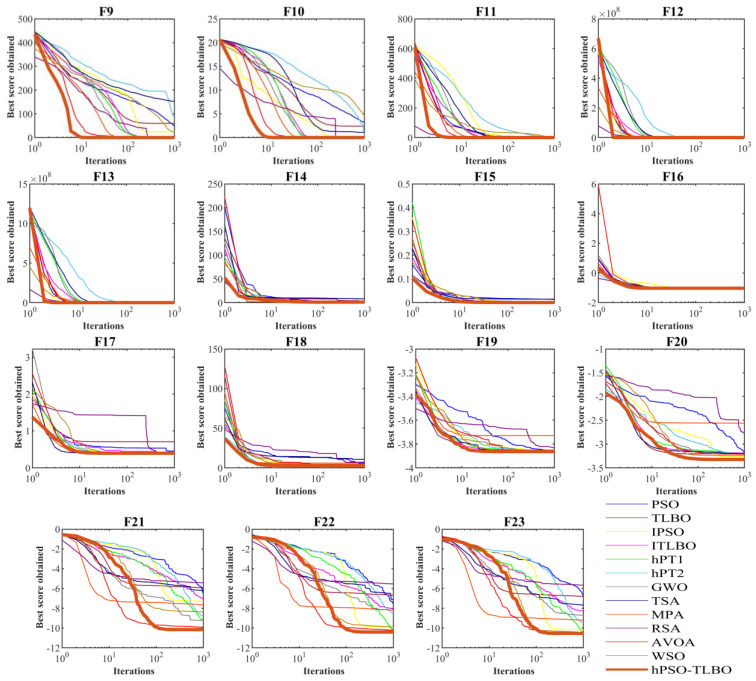
Convergence curves of performance hPSO-TLBO and twelve competitor optimizers on functions F1 to F23.

Multimodal functions with a fixed number of dimensions are suitable criteria for simultaneous measurement of exploration and exploitation in metaheuristic algorithms. Table 3 presents the outcomes achieved by applying hPSO-TLBO and other competing optimizers to fixed-dimension multimodal benchmarks F14 to F23. The proposed hPSO-TLBO emerged as the top-performing optimizer for functions F14 to F23, showcasing its effectiveness. In cases where hPSO-TLBO shares the same mean index values with certain competing algorithms, its superior performance is evident through better std index values. The simulation results highlight hPSO-TLBO’s exceptional balance between exploration and exploitation, surpassing competing algorithms in handling fixed-dimension multimodal functions F14 to F23. The performance comparison by convergence curves is illustrated in Figure 2.

### 4.5. Evaluation CEC 2017 Test Suite

**Figure 3:**
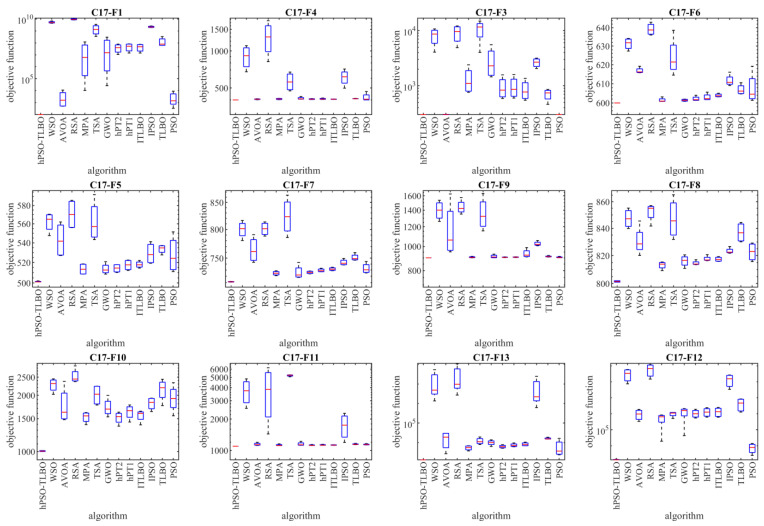

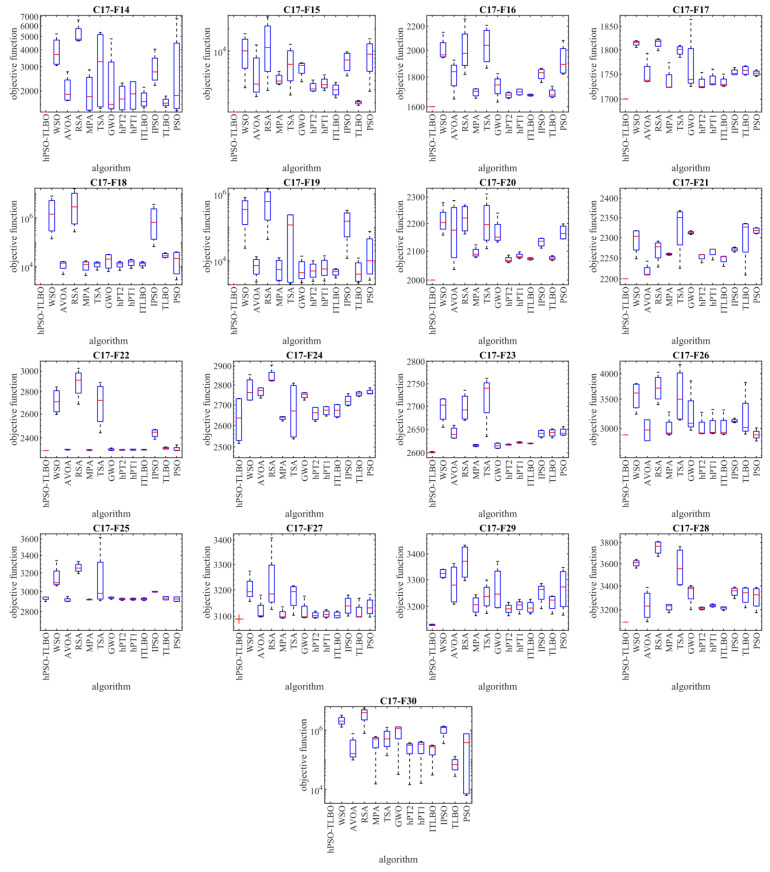
Boxplot diagram of the hPSO-TLBO and competitor optimizers’ performances on the CEC 2017 test set.

In this subsection, the performance of hPSO-TLBO is evaluated in handling the CEC 2017 test suite. The test suite employed in this study comprises thirty standard benchmarks, including three unimodal functions (C17-F1 to C17-F3), seven multimodal functions (C17-F4 to C17-F10), ten hybrid functions (C17-F11 to C17-F20), and ten composition functions (C17-F21 to C17-F30). However, the C17-F2 function was excluded from the simulations due to its unstable behavior. Detailed CEC 2017 test suite information can be found in [89]. The implementation results of hPSO-TLBO and other competing algorithms on the CEC 2017 test suite are presented in Table 4. Boxplots of the performance of metaheuristic methods in handling benchmarks from the CEC 2017 set are shown in Figure 3. The optimization results demonstrate that hPSO-TLBO emerged as the top-performing optimizer for functions C17-F1, C17-F3 to C17-F24, and C17-F26 to C17-F30. Overall, evaluating the benchmark functions in the CEC 2017 test set revealed that the proposed hPSO-TLBO approach outperforms competing algorithms in achieving superior results.

### 4.6. Statistical Analysis

To assess the statistical significance of the superiority of the proposed hPSO-TLBO approach over competing algorithms, a nonparametric statistical test, namely the Wilcoxon signed-rank test [91], was conducted in this subsection. The test examines the mean differences between two data samples and determines whether they differ significantly. The obtained p-values from the test were used to evaluate the significance of the differences between hPSO-TLBO and the competing algorithms. The results of the Wilcoxon signed-rank test, indicating the significance of the performance differences among the metaheuristic algorithms, are presented in Table 5. The statistical analysis reveals that the proposed hPSO-TLBO approach exhibits a significant statistical advantage over the competing algorithms when the p-value is less than 0.05. The Wilcoxon signed-rank test notably confirms that hPSO-TLBO outperforms all twelve competing metaheuristic algorithms with a significant statistical advantage.

## 5. hPSO-TLBO for Real-World Applications

In this section, we examine the effectiveness of the proposed hPSO-TLBO approach in addressing four engineering design problems, highlighting one of the key applications of metaheuristic algorithms. These algorithms play a crucial role in solving optimization problems in real-world scenarios.

### 5.1. Pressure Vessel Design Problem

**Figure 4:**
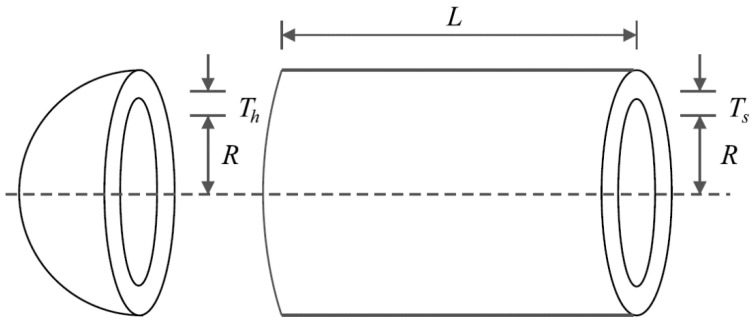
Schematic of pressure vessel design.

The design of a pressure vessel poses a significant engineering challenge, requiring careful consideration and analysis. The primary objective of this design is to achieve the minimum construction cost while meeting all necessary specifications and requirements. To provide a visual representation, Figure 4 depicts the schematic of the pressure vessel design, aiding in understanding its structural elements and overall layout. The mathematical model governing the pressure vessel design is presented below. This model encapsulates the equations and parameters that define the behavior and characteristics of the pressure vessel [92]:

Consider:

$$X=\left[x_{1},x_{2},x_{3},x_{4}\right]=\left[T_{s},T_{h},R,L\right].$$

Minimize:

$$f\left(x\right)=0.6224x_{1}x_{3}x_{4}+1.778x_{2}x_{3}^{2}+3.1661x_{1}^{2}x_{4}+19.84x_{1}^{2}x_{3}.$$

Subject to:

$$g_{1}\left(x\right)=-x_{1}+0.0193x_{3}\leq 0,$$

$$g_{2}\left(x\right)=-x_{2}+0.00954x_{3}\leq 0,$$

$$g_{3}\left(x\right)=-\pi x_{3}^{2}x_{4}-\frac{4}{3}\pi x_{3}^{3}+1296000\leq 0,$$

$$g_{4}\left(x\right)=x_{4}-240\leq 0.$$

with

$$0\leq x_{1},x_{2}\leq 100{\;\mathrm{and}\;10\leq x}_{3},x_{4}\leq 200.$$

**Figure 5:**
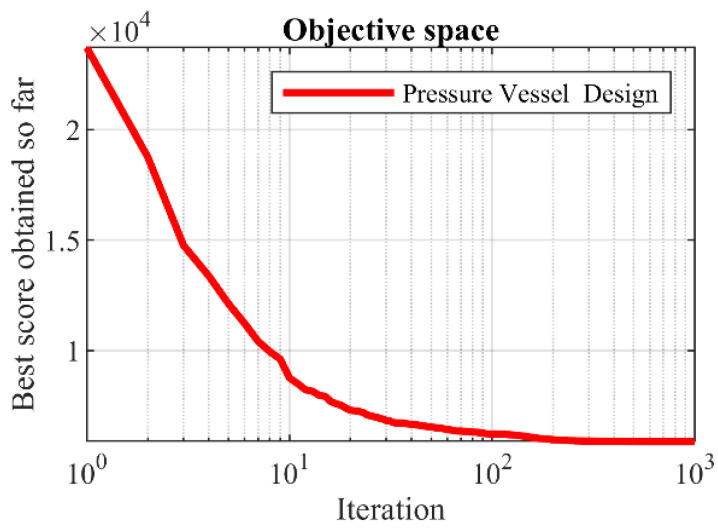
hPSO-TLBO’s performance convergence curve on pressure vessel design.

The results of employing hPSO-TLBO and competing algorithms to optimize pressure vessel design are presented in Table 6 and Table 7. The results obtained from the analysis indicate that the hPSO-TLBO algorithm successfully achieved the optimal design solution for the pressure vessel. The design variables were determined as (0.7780271, 0.3845792, 40.312284, 200), with the objective function value of 5882.9013. Furthermore, a comprehensive evaluation of the simulation results reveals that the hPSO-TLBO algorithm outperforms other competing algorithms regarding statistical indicators for the pressure vessel design problem. This superiority is demonstrated by the ability of hPSO-TLBO to deliver more favorable results. To visualize the convergence of the hPSO-TLBO algorithm towards the optimal design, Figure 5 illustrates the convergence curve associated with achieving the optimal solution for the pressure vessel.

### 5.2. Speed Reducer Design Problem

**Figure 6:**
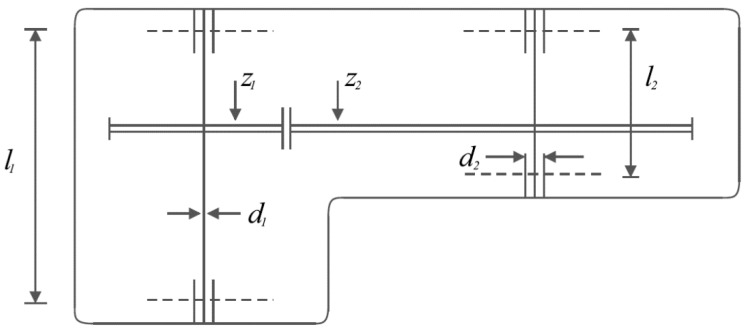
Schematic of speed reducer design.

The speed reducer design is a real-world application in engineering to minimize speed reducer weight. The speed reducer design schematic is shown in Figure 6. As expressed in [93,94], the mathematical model for the design of the speed reducer is given by the following equation and constraints:

Consider:

$$X=\left[x_{1,}x_{2},x_{3},x_{4},x_{5}{,x}_{6},x_{7}\right]=\left[b,m,p,l_{1},l_{2},d_{1},d_{2}\right].$$

Minimize:

$$f\left(x\right)=0.7854x_{1}x_{2}^{2}\left(3.3333x_{3}^{2}+14.9334x_{3}-43.0934\right)-1.508x_{1}\left(x_{6}^{2}+x_{7}^{2}\right)+7.4777\left(x_{6}^{3}+x_{7}^{3}\right)+0.7854(x_{4}x_{6}^{2}+x_{5}x_{7}^{2}).$$

Subject to:

$$g_{1}\left(x\right)=\frac{27}{x_{1}x_{2}^{2}x_{3}}-1\;\leq \;0,\;g_{2}\left(x\right)=\frac{397.5}{x_{1}x_{2}^{2}x_{3}}-1\leq \;0,$$

$$g_{3}\left(x\right)=\frac{1.93x_{4}^{3}}{x_{2}x_{3}x_{6}^{4}}-1\leq \;0,\;g_{4}\left(x\right)=\frac{1.93x_{5}^{3}}{x_{2}x_{3}x_{7}^{4}}-1\;\leq \;0,$$

$$g_{5}\left(x\right)=\frac{1}{110x_{6}^{3}}\sqrt{{\left(\frac{745x_{4}}{x_{2}x_{3}}\right)}^{2}+16.9\cdot 10^{6}}-1\leq \;0,$$

$$g_{6}(x)=\frac{1}{85x_{7}^{3}}\sqrt{{\left(\frac{745x_{5}}{x_{2}x_{3}}\right)}^{2}+157.5\cdot 10^{6}}-1\;\leq \;0,$$

$$g_{7}\left(x\right)=\frac{x_{2}x_{3}}{40}-1\;\leq \;0,\;g_{8}\left(x\right)=\frac{{5x}_{2}}{x_{1}}-1\;\leq \;0,$$

$$g_{9}\left(x\right)=\frac{x_{1}}{12x_{2}}-1\;\leq \;0,$$

$$g_{10}\left(x\right)=\frac{{1.5x}_{6}+1.9}{x_{4}}-1\;\leq \;0,$$

$$g_{11}\left(x\right)=\frac{{1.1x}_{7}+1.9}{x_{5}}-1\;\leq \;0.$$

with

$$2.6\leq x_{1}\leq 3.6,\;0.7\leq x_{2}\leq 0.8,\;17\leq x_{3}\leq 28,$$

$$7.3\leq x_{4}\leq 8.3,\;7.8\leq x_{5}\leq 8.3,\;2.9\leq x_{6}\leq 3.9,$$

and

$$5\leq x_{7}\leq 5.5.$$

**Figure 7:**
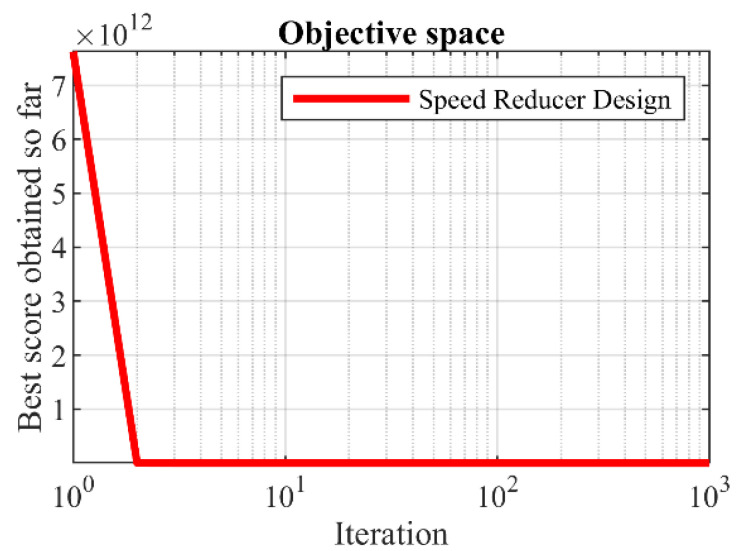
hPSO-TLBO’s performance convergence curve on speed reducer design.

Table 8 and Table 9 display the outcomes obtained by applying the hPSO-TLBO algorithm and other competing algorithms to optimize the design of the speed reducer. The obtained results demonstrate that the hPSO-TLBO algorithm successfully generated the optimal design solution for the speed reducer. The model variables were determined as (3.5, 0.7, 17, 7.3, 7.8, 3.3502147, 5.2866832), resulting in an objective function value of 2996.3482. The simulation results clearly indicate that hPSO-TLBO performs better than other competing methods in tackling the speed reducer design problem. Furthermore, it consistently produces better outcomes and achieves improved results. Figure 7 portrays the convergence curve of the hPSO-TLBO algorithm as it progresses toward attaining the optimal design for the speed reducer, providing a visual representation of its successful performance.

### 5.3. Welded Beam Design

**Figure 8:**
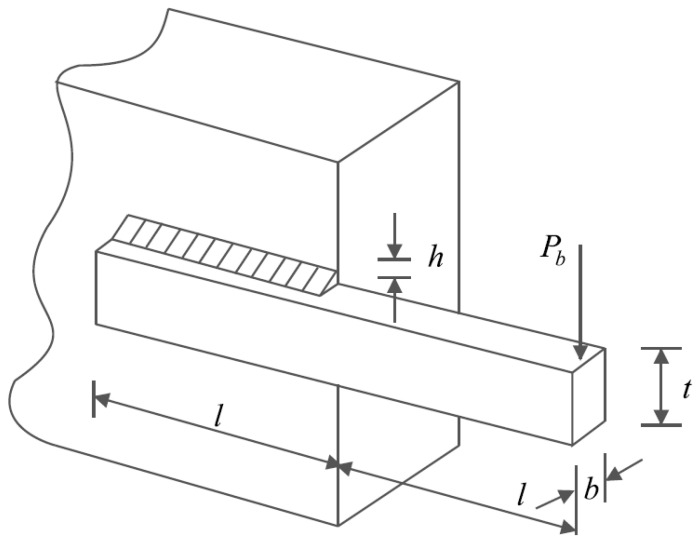
Schematic of welded beam design.

The design of welded beams holds significant importance in real-world engineering applications. Its primary objective is to minimize the fabrication cost associated with welded beam design. To aid in visualizing the design, Figure 8 presents the schematic of a welded beam, illustrating its structural configuration and critical elements. The mathematical model to analyze and optimize the welded beam design is as follows [16]:

Consider:

$$X=\left[x_{1},x_{2},x_{3},x_{4}\right]=\left[h,l,t,b\right].$$

Minimize:

$$f(x)=1.10471x_{1}^{2}x_{2}+0.04811x_{3}x_{4}(14.0+x_{2}).$$

Subject to:

$$g_{1}\left(x\right)=\tau \left(x\right)-13600\;\leq \;0,$$

$$g_{2}\left(x\right)=\sigma \left(x\right)-30000\;\leq \;0,$$

$$g_{3}\left(x\right)=x_{1}-x_{4}\leq \;0,$$

$$g_{4}\left(x\right)=0.10471x_{1}^{2}+0.04811x_{3}x_{4}\;(14+x_{2})-5.0\;\leq \;0,$$

$$g_{5}\left(x\right)=0.125-x_{1}\leq \;0,$$

$$g_{6}\left(x\right)=\delta \;\left(x\right)-0.25\;\leq \;0,$$

$$g_{7}\left(x\right)=6000-p_{c}\;\left(x\right)\leq \;0.$$

where

$$\tau \left(x\right)=\sqrt{{\left(\tau^{\prime }\right)}^{2}+\left(2\tau \tau^{\prime }\right)\frac{x_{2}}{2R}+{\left(\tau^{\prime \prime }\right)}^{2}\;},\;\tau^{\prime }=\frac{6000}{\sqrt{2}x_{1}x_{2}},\;\tau^{\prime \prime }=\frac{MR}{J},$$

$$M=6000\left(14+\frac{x_{2}}{2}\right),\;R=\sqrt{\frac{x_{2}^{2}}{4}+{\left(\frac{x_{1}+x_{3}}{2}\right)}^{2}},$$

$$J=2\sqrt{2}x_{1}x_{2}\left(\frac{x_{2}^{2}}{12}+{\left(\frac{x_{1}+x_{3}}{2}\right)}^{2}\right),\;\sigma \left(x\right)=\frac{504000}{x_{4}x_{3}^{2}},$$

$$\delta \;\left(x\right)=\frac{65856000}{\left(30\cdot 10^{6}\right)x_{4}x_{3}^{3}},$$

$$p_{c}\;\left(x\right)=\frac{4.013\left(30\cdot 10^{6}\right)x_{3}x_{4}^{3}}{1176}\left(1-\frac{x_{3}}{28}\sqrt{\frac{30\cdot 10^{6}}{4(12\cdot 10^{6})}}\right).$$

with

$$0.1\leq x_{1},\;x_{4}\leq 2\;\mathrm{and}\;0.1\leq x_{2},\;x_{3}\leq 10.$$

**Figure 9:**
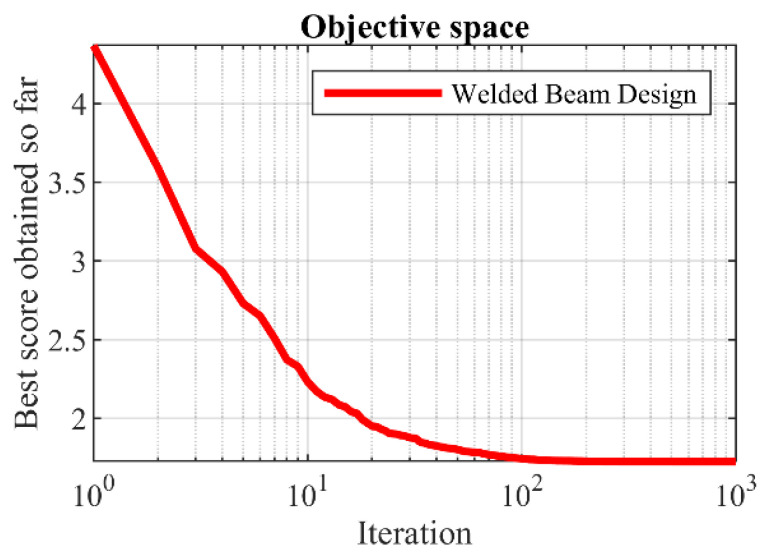
hPSO-TLBO’s performance convergence curve on welded beam design.

The optimization results for the welded beam design, achieved by employing the proposed hPSO-TLBO algorithm and other competing optimizers, are presented in Table 10 and Table 11. The proposed hPSO-TLBO algorithm yielded the optimal design for the welded beam, as indicated by the obtained results. The design variables were determined to have values of (0.2057296, 3.4704887, 9.0366239, 0.2057296), and the corresponding objective function value was found to be 1.7248523. The simulation outcomes demonstrate that hPSO-TLBO outperforms competing algorithms in terms of statistical indicators and overall effectiveness in optimizing the welded beam design. The process of achieving the optimal design using hPSO-TLBO for the welded beam is depicted in Figure 9.

### 5.4. Tension/Compression Spring Design

**Figure 10:**
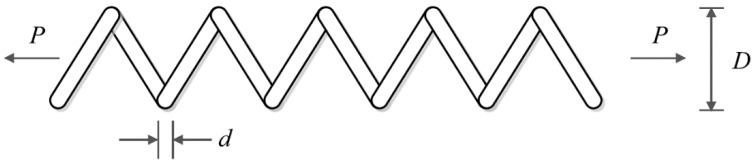
Schematic of tension/compression spring design.

The tension/compression spring design is an optimization problem in real-world applications to minimize the weight of a tension/compression spring. The tension/compression spring design schematic is shown in Figure 10. The following mathematical model represents a tension/compression spring, as outlined in [16]:

Consider:

$$X=\left[x_{1},x_{2},x_{3}\right]=\left[d,D,P\right].$$

Minimize:

$$f\left(x\right)=\left(x_{3}+2\right)x_{2}x_{1}^{2}.$$

Subject to:

$$g_{1}\left(x\right)=1-\frac{x_{2}^{3}x_{3}}{71785x_{1}^{4}}\;\leq \;0,$$

$$g_{2}\left(x\right)=\frac{4x_{2}^{2}-x_{1}x_{2}}{12566(x_{2}x_{1}^{3})}+\frac{1}{5108x_{1}^{2}}-1\leq \;0,$$

$$g_{3}\left(x\right)=1-\frac{140.45x_{1}}{x_{2}^{2}x_{3}}\leq \;0,\;{\;g}_{4}\left(x\right)=\frac{x_{1}+x_{2}}{1.5}-1\;\leq \;0$$

with

$$0.05\leq x_{1}\leq 2,\;{0.25\leq x}_{2}\leq 1.3\;and\;2\leq \;x_{3}\leq 15.$$

**Figure 11:**
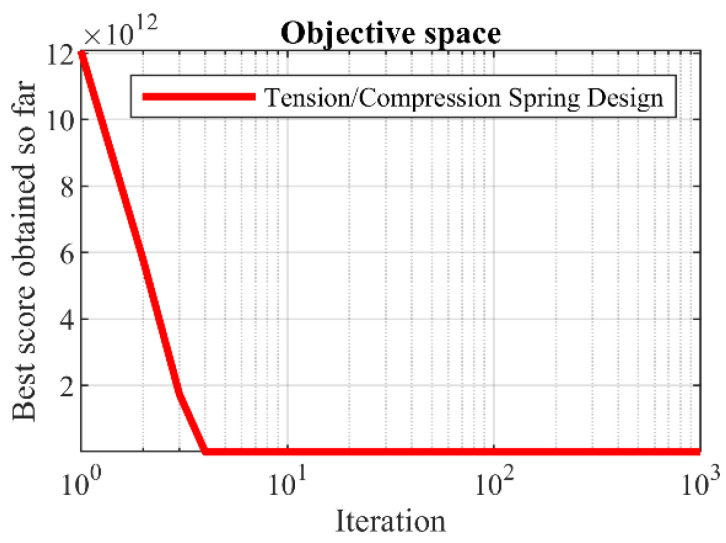
hPSO-TLBO’s performance convergence curve on tension/compression spring.

Table 12 and Table 13 showcase the results obtained when employing the hPSO-TLBO algorithm and other competing algorithms for the optimization of the tension/compression spring design. The proposed hPSO-TLBO approach yielded the optimal design for the tension/compression spring, as evidenced by the obtained results. The design variables were determined to have values of (0.0516891, 0.3567177, 11.288966), and the corresponding value of the objective function was found to be 0.0126652. Simulation outcomes demonstrate that hPSO-TLBO outperforms competing algorithms, delivering superior outcomes in addressing the tension/compression spring problem. The convergence curve of hPSO-TLBO, illustrating its ability to achieve the optimal design for a tension/compression spring, is depicted in Figure 11.

## 6. Conclusions and Future Works

This paper presented a novel hybrid metaheuristic algorithm called hPSO-TLBO, which combines the strengths of particle swarm optimization (PSO) and teaching–learning-based optimization (TLBO). The integration of PSO’s exploitation capability with TLBO’s exploration ability forms the foundation of hPSO-TLBO. The performance of hPSO-TLBO was evaluated on a diverse set of optimization tasks, including fifty-two standard benchmark functions and the CEC 2017 test suite. The results showcase the favorable performance of hPSO-TLBO across a range of benchmark functions, highlighting its capability to balance exploration and exploitation strategies effectively. A comparative analysis with twelve established metaheuristic algorithms further confirms the superior performance of hPSO-TLBO, which is statistically significant according to Wilcoxon analysis. Additionally, the successful application of hPSO-TLBO in solving four engineering design problems showcased its efficacy in real-world scenarios.

The introduction of hPSO-TLBO opens up several avenues for future research. One promising direction involves developing discrete or multi-objective versions of hPSO-TLBO. Exploring the application of hPSO-TLBO in diverse real-world problem domains is another great research prospect.

## Figures and Tables

**Table 1:** Optimization results of unimodal functions.

F		hPSO-TLBO	WSO	AVOA	RSA	MPA	TSA	GWO	hPT2	hPT1	ITLBO	IPSO	TLBO	PSO
F1	Mean	0	58.07159	7.09E-61	7.09E-61	1.69E-49	4.1E-47	7.09E-61	0.131844	1.63E-59	7.09E-61	1.17E-16	0.088953	26.87535
	Best	0	4.665568	5.99E-63	5.99E-63	3.36E-52	1.27E-50	5.99E-63	0.092965	1.38E-61	5.99E-63	4.72E-17	0.000429	15.79547
	Worst	0	210.5042	3.09E-60	3.09E-60	1.46E-48	2.91E-46	3.09E-60	0.177363	7.11E-59	3.09E-60	3.29E-16	1.231554	50.15932
	Std	0	45.40585	8.38E-61	8.38E-61	3.38E-49	8.61E-47	8.38E-61	0.023884	1.92E-59	8.38E-61	6.16E-17	0.267405	9.002594
	Median	0	40.01959	4.31E-61	4.31E-61	3.67E-50	3.77E-48	4.31E-61	0.13263	9.91E-60	4.31E-61	9.97E-17	0.008564	24.84615
	Rank	1	11	2	2	5	6	2	9	4	3	7	8	10
F2	Mean	0	1.885416	5.43E-36	5.43E-36	6.14E-28	1.86E-28	5.43E-36	0.228358	1.25E-34	5.44E-36	4.83E-08	0.789031	2.456858
	Best	0	0.583709	1.95E-37	1.95E-37	1.63E-29	1.78E-30	1.95E-37	0.141042	4.49E-36	1.97E-37	3.07E-08	0.039898	1.537836
	Worst	0	6.560237	3.17E-35	3.17E-35	4.15E-27	1.61E-27	3.17E-35	0.32117	7.29E-34	3.17E-35	1.09E-07	2.196863	3.353961
	Std	0	1.526648	7.67E-36	7.67E-36	9.41E-28	4.55E-28	7.67E-36	0.0542	1.76E-34	7.67E-36	1.61E-08	0.621855	0.468754
	Median	0	1.348491	2.61E-36	2.61E-36	3.1E-28	1.74E-29	2.61E-36	0.236442	5.99E-35	2.63E-36	4.52E-08	0.514708	2.415588
	Rank	1	10	2	2	6	5	2	8	4	3	7	9	11
F3	Mean	0	1573.92	8.72E-16	8.72E-16	2.21E-12	1.04E-10	17586.09	14.07412	2E-14	8.72E-16	418.9635	341.9832	1911.093
	Best	0	916.7392	9.45E-21	9.45E-21	1.62E-16	2.18E-18	1819.369	5.263941	2.17E-19	9.48E-21	216.719	19.18004	1254.853
	Worst	0	3121.842	1.62E-14	1.62E-14	1.27E-11	1.72E-09	30564.02	43.12089	3.73E-13	1.62E-14	1045.265	903.4752	3047.672
	Std	0	540.174	3.53E-15	3.53E-15	3.77E-12	3.75E-10	7362.857	9.262445	8.11E-14	3.53E-15	189.5394	248.1753	550.4119
	Median	0	1373.011	1.87E-17	1.87E-17	1.61E-13	9.48E-14	17907.73	10.46683	4.3E-16	1.87E-17	352.7354	258.2018	1850.929
	Rank	1	10	2	2	5	6	12	7	4	3	9	8	11
F4	Mean	0	15.23952	4.92E-16	4.92E-16	4.92E-16	0.003897	45.65984	0.482066	1.13E-14	4.92E-16	1.088936	5.533212	2.492984
	Best	0	10.49816	2.63E-17	2.63E-17	2.67E-17	8.51E-05	0.797019	0.234307	6.04E-16	2.63E-17	8.72E-09	2.017958	1.952933
	Worst	0	21.00169	2.3E-15	2.3E-15	2.3E-15	0.031568	80.80556	0.848542	5.29E-14	2.3E-15	4.341798	11.77172	3.518006
	Std	0	2.484433	5.71E-16	5.71E-16	5.71E-16	0.006836	25.48147	0.165382	1.31E-14	5.71E-16	1.193547	2.153131	0.401768
	Median	0	15.65954	2.55E-16	2.55E-16	2.55E-16	0.001295	48.83455	0.467904	5.85E-15	2.55E-16	0.799113	5.183052	2.452526
	Rank	1	11	2	2	4	6	12	7	5	3	8	10	9
F5	Mean	0	9516.431	1.066232	12.51932	21.61701	26.15764	25.12883	85.84715	24.48729	24.6691	39.87864	4064.647	525.661
	Best	0	1188.169	1.025518	1.025505	21.1648	23.70648	24.59573	25.39379	23.55223	23.59366	23.85439	24.20836	202.6519
	Worst	0	81,693.13	1.089267	26.63229	22.24073	26.54457	26.40645	334.024	25.01649	26.42328	148.4471	79368.29	1989.749
	Std	0	17,267.51	0.020618	12.68763	0.333573	0.674837	0.500437	87.30341	0.473488	0.806602	38.14302	17309.01	365.6698
	Median	0	4943.759	1.052191	1.088766	21.59754	26.44754	24.93124	27.53712	24.16495	24.2643	24.28404	76.94997	420.079
	Rank	1	13	2	3	4	8	7	10	5	6	9	12	11
F6	Mean	0	88.93565	0.026507	5.716556	0.026507	3.27064	0.098382	0.159556	0.608782	1.137933	0.026507	0.08241	30.11387
	Best	0	14.95731	0.009897	3.257687	0.009897	2.279437	0.023498	0.089817	0.22729	0.233809	0.009897	0.010145	13.77592
	Worst	0	337.0663	0.05023	6.428149	0.05023	4.238575	0.308096	0.250358	1.153614	1.917394	0.05023	0.497324	55.34426
	Std	0	82.15401	0.01201	0.885657	0.01201	0.596429	0.084744	0.041251	0.275827	0.423887	0.01201	0.125926	11.65958
	Median	0	61.32398	0.029174	6.111534	0.029174	3.382167	0.060521	0.16307	0.670012	1.099359	0.029174	0.030784	27.94543
	Rank	1	13	4	11	3	10	6	7	8	9	2	5	12
F7	Mean	2.54E-05	0.000115	9.04E-05	6.18E-05	0.000517	0.003862	0.001161	0.010269	0.000767	0.001383	0.046565	0.162282	0.009365
	Best	2.35E-06	2.3E-05	1.5E-05	1.43E-05	0.000133	0.001351	8.11E-05	0.00354	0.000168	8.83E-05	0.012474	0.060852	0.002701
	Worst	6.89E-05	0.000317	0.000261	0.000159	0.000801	0.008816	0.004798	0.019927	0.001803	0.002604	0.08422	0.362473	0.019393
	Std	1.93E-05	7.92E-05	6.36E-05	3.04E-05	0.000184	0.002009	0.001241	0.004336	0.00042	0.000755	0.021471	0.067986	0.004146
	Median	1.83E-05	8.37E-05	7.21E-05	5.91E-05	0.000502	0.003319	0.000752	0.010003	0.00078	0.001351	0.045702	0.156642	0.009006
	Rank	1	4	3	2	5	9	7	11	6	8	12	13	10
Sum rank		7	72	17	24	32	50	48	59	36	35	54	65	74
Mean rank		1	10.28571	2.428571	3.428571	4.571429	7.142857	6.857143	8.428571	5.142857	5	7.714286	9.285714	10.57143
Total ranking		1	12	2	3	4	8	7	10	6	5	9	11	13

**Table 2:** Optimization results of high-dimensional multimodal functions.

F		hPSO-TLBO	WSO	AVOA	RSA	MPA	TSA	GWO	hPT2	hPT1	ITLBO	IPSO	TLBO	PSO
F8	Mean	−12,498.6	−7441.49	−12,216.6	−6018.45	−9764.22	−6637.83	−10,978.1	−8130.21	−6585.37	−6161.34	−3679.16	−6997.53	−8648.79
	Best	−12,622.8	−9148.22	−12,328.4	−6214.34	−10450.1	−7689.05	−12314	−9292.31	−7292.22	−7424.16	−4727.93	−8481.16	−9748.05
	Worst	−11,936.3	−6604.3	−11715	−5570.82	−9263.23	−5103.76	−8038.63	−7294.3	−5644.31	−5239.76	−3087.21	−5627.46	−7433.78
	Std	185.933	632.4496	166.3366	191.7651	309.4905	620.8402	1488.781	623.0539	425.9794	530.8499	431.4128	643.493	548.4352
	Median	−12,577.8	−7384.82	−12,293.4	−6058.43	−9804.06	−6608.26	−11,853.8	−8022.38	−6586.24	−6192.06	−3598.29	−7140.36	−8616.57
	Rank	1	7	2	12	4	9	3	6	10	11	13	8	5
F9	Mean	0	21.70166	6.84E-16	6.84E-16	6.84E-16	152.5399	6.84E-16	86.19788	1.57E-14	6.84E-16	25.11628	59.66324	48.1797
	Best	0	12.88139	0	0	0	79.07429	0	46.51055	0	0	12.27323	35.06638	20.47009
	Worst	0	40.48713	4.56E-15	4.56E-15	4.56E-15	253.9196	4.56E-15	131.5313	1.05E-13	4.56E-15	42.95628	100.9408	67.75744
	Std	0	7.415334	1.27E-15	1.27E-15	1.27E-15	43.88833	1.27E-15	21.68033	2.92E-14	1.27E-15	7.886816	16.21153	11.8805
	Median	0	19.99106	0	0	0	146.858	0	85.53992	0	0	23.23147	57.33199	46.35864
	Rank	1	4	2	2	2	9	2	8	3	2	5	7	6
F10	Mean	8.88E-16	4.662244	1.52E-15	1.52E-15	4.5E-15	1.094762	4.34E-15	0.509188	1.55E-14	4.65E-15	7.24E-09	2.402969	3.150025
	Best	8.88E-16	2.980712	1.17E-15	1.17E-15	1.74E-15	8E-15	1.46E-15	0.088639	7.43E-15	4.3E-15	4.11E-09	1.4921	2.5393
	Worst	8.88E-16	7.22389	1.74E-15	1.74E-15	4.87E-15	2.972354	8E-15	2.216136	2.05E-14	4.87E-15	1.27E-08	4.455792	4.090043
	Std	0	1.050992	1.39E-16	1.39E-16	6.46E-16	1.350455	1.96E-15	0.582673	3.19E-15	1.39E-16	2.01E-09	0.738078	0.341285
	Median	8.88E-16	4.563645	1.46E-15	1.46E-15	4.59E-15	2.03E-14	4.59E-15	0.171211	1.4E-14	4.59E-15	6.81E-09	2.408861	3.198028
	Rank	1	12	2	2	4	9	3	8	6	5	7	10	11
F11	Mean	0	1.512162	5.37E-05	5.37E-05	5.37E-05	0.007845	5.37E-05	0.352208	0.001234	5.37E-05	6.351044	0.163292	1.298331
	Best	0	0.972628	0	0	0	0	0	0.22393	0	0	2.639784	0.002841	1.134942
	Worst	0	2.894179	0.000755	0.000755	0.000755	0.018104	0.000755	0.472258	0.017341	0.000755	11.13516	0.771711	1.520656
	Std	0	0.466901	0.000176	0.000176	0.000176	0.005425	0.000176	0.070424	0.004033	0.000176	2.341121	0.196554	0.106538
	Median	0	1.410629	0	0	0	0.008084	0	0.367154	0	0	6.442223	0.107808	1.275578
	Rank	1	8	2	2	2	4	2	6	3	2	9	5	7
F12	Mean	1.57E-32	2.882539	0.0016	1.162552	0.0016	5.105635	0.019307	0.807492	0.036737	0.064448	0.186664	1.324184	0.243809
	Best	1.57E-32	0.841956	0.000504	0.678786	0.000504	0.915241	0.002818	0.001689	0.011573	0.022023	0.001228	0.002242	0.055508
	Worst	1.57E-32	6.513307	0.003481	1.450986	0.003481	12.45602	0.121128	3.392312	0.079946	0.120129	0.821731	4.600176	0.576584
	Std	2.74E-48	1.574216	0.000836	0.26155	0.000836	3.338511	0.03409	1.029815	0.019191	0.018042	0.264223	1.106087	0.119679
	Median	1.57E-32	2.551467	0.001521	1.225406	0.001521	3.794602	0.006401	0.371998	0.034924	0.063014	0.072401	1.133776	0.234452
	Rank	1	12	3	10	2	13	4	9	5	6	7	11	8
F13	Mean	1.35E-32	3171.704	0.02061	0.02061	0.022811	2.414466	0.209698	0.049488	0.473338	0.991581	0.070534	3.199289	2.406486
	Best	1.35E-32	12.16954	1.88E-06	1.88E-06	0.007909	1.790394	0.047447	0.019729	4.32E-05	0.539453	0.007909	0.028472	1.150151
	Worst	1.35E-32	54770.43	0.03811	0.03811	0.03811	3.308616	0.624983	0.105229	0.875262	1.377995	0.844427	11.10643	3.48383
	Std	2.74E-48	11920.44	0.010099	0.010099	0.009378	0.481365	0.159473	0.023458	0.231945	0.200568	0.179163	2.605342	0.652146
	Median	1.35E-32	38.99428	0.020744	0.020744	0.023381	2.251915	0.166636	0.04162	0.476406	1.000383	0.028803	2.930893	2.536779
	Rank	1	13	3	2	4	11	7	5	8	9	6	12	10
Sum Rank		6	56	14	30	18	55	21	42	35	35	47	53	47
Mean rank		1	9.333333	2.333333	5	3	9.166667	3.5	7	5.833333	5.833333	7.833333	8.833333	7.833333
Total ranking		1	11	2	5	3	10	4	7	6	6	8	9	8

**Table 3:** Optimization results of fixed-dimensional multimodal functions.

F		hPSO-TLBO	WSO	AVOA	RSA	MPA	TSA	GWO	hPT2	hPT1	ITLBO	IPSO	TLBO	PSO
F14	Mean	0.397887	0.397928	0.397928	0.409123	0.398381	0.39796	0.397928	0.397928	0.397929	0.397992	0.397928	0.703449	0.457962
	Best	0.397887	0.397887	0.397887	0.39867	0.397887	0.397893	0.397887	0.397887	0.397888	0.397899	0.397887	0.397887	0.397887
	Worst	0.397887	0.398145	0.398145	0.474877	0.401023	0.398168	0.398146	0.398145	0.398145	0.398164	0.398145	2.506628	1.591156
	Std	0	7.35E-05	7.36E-05	0.016725	0.000896	8.73E-05	7.35E-05	7.36E-05	7.35E-05	8.56E-05	7.36E-05	0.61029	0.260471
	Median	0.397887	0.397894	0.397894	0.403092	0.397971	0.397918	0.397895	0.397894	0.397894	0.397973	0.397894	0.397918	0.397966
	Rank	1	4	2	10	9	7	5	3	6	8	2	12	11
F15	Mean	3	3.2491	3.249101	5.693971	6.034843	10.74003	3.249123	3.249101	3.249112	3.249101	3.2491	3.2491	7.040393
	Best	3	3.001098	3.001098	3.002107	3.013375	3.001104	3.001098	3.001098	3.001101	3.001099	3.001098	3.001098	3.003001
	Worst	3	5.127366	5.127366	27.95563	28.91822	81.45687	5.127367	5.127367	5.127377	5.127368	5.127366	5.127366	31.20499
	Std	1.14E-15	0.489279	0.489279	7.321184	5.961004	22.46757	0.489272	0.489279	0.489277	0.489279	0.489279	0.489279	8.990184
	Median	3	3.04441	3.04441	3.14002	3.541047	3.140014	3.044417	3.04441	3.04443	3.044411	3.04441	3.04441	3.179816
	Rank	1	2	6	10	11	13	9	5	8	7	4	3	12
F16	Mean	−3.86278	−3.85185	−3.85185	−3.82907	−3.7303	−3.8515	−3.84977	−3.85185	−3.85051	−3.85088	−3.85185	−3.85185	−3.85171
	Best	−3.86278	−3.86278	−3.86278	−3.85382	−3.86278	−3.86268	−3.86277	−3.86278	−3.86278	−3.86253	−3.86278	−3.86278	−3.86276
	Worst	−3.86278	−3.81789	−3.81789	−3.77776	−3.31594	−3.81781	−3.8175	−3.81789	−3.81778	−3.81766	−3.81789	−3.81789	−3.81759
	Std	2.22E-15	0.010564	0.010564	0.021113	0.12881	0.010411	0.010321	0.010564	0.010614	0.01013	0.010564	0.010564	0.010686
	Median	−3.86278	−3.85184	−3.85184	−3.83257	−3.73109	−3.8518	−3.85033	−3.85184	−3.85132	−3.85144	−3.85184	−3.85184	−3.85177
	Rank	1	2	4	11	12	7	10	5	9	8	3	3	6
F17	Mean	−3.322	−3.24156	−3.21013	−2.76672	−2.56172	−3.19829	−3.19374	−3.21528	−3.20179	−3.18745	−3.25727	−3.20672	−3.17472
	Best	−3.322	−3.31434	−3.28525	−3.038	−3.22873	−3.31233	−3.30934	−3.31434	−3.31434	−3.29852	−3.31434	−3.31434	−3.2439
	Worst	−3.322	−3.15104	−3.10447	−1.75378	−1.84535	−3.07187	−3.0508	−3.096	−3.00597	−2.93767	−3.20079	−3.04679	−2.9906
	Std	4.34E-16	0.044955	0.059342	0.276987	0.31673	0.060395	0.075584	0.064426	0.079772	0.082864	0.026923	0.077408	0.06172
	Median	−3.322	−3.2565	−3.21667	−2.84296	−2.61407	−3.18964	−3.20415	−3.24865	−3.21227	−3.19193	−3.2616	−3.23928	−3.18554
	Rank	1	3	5	12	13	8	9	4	7	10	2	6	11
F18	Mean	−10.1532	−8.37918	−9.91819	−5.42633	−7.63223	−6.1929	−9.24171	−8.80122	−9.24604	−7.01014	−7.31095	−5.92734	−6.48809
	Best	−10.1532	−10.1447	−10.1531	−5.6612	−10.1516	−10.1238	−10.1524	−10.153	−10.1529	−9.25331	−10.1531	−10.0716	−9.60167
	Worst	−10.1532	−3.1694	−9.54887	−5.05701	−5.05701	−3.10699	−5.28384	−5.25966	−5.09691	−3.88379	−3.1694	−3.14741	−2.90764
	Std	2.03E-15	2.727104	0.169179	0.169179	1.920128	2.796647	1.602728	1.93072	1.67125	1.762604	2.999827	2.451367	2.42999
	Median	−10.1532	−9.84425	−9.95037	−5.45851	−7.99154	−5.27858	−9.84367	−9.80279	−9.91216	−7.29137	−9.75152	−5.33906	−7.07368
	Rank	1	6	2	13	7	11	4	5	3	9	8	12	10
F19	Mean	−10.4029	−9.8836	−10.2207	−5.53737	−8.18247	−7.12047	−8.19905	−8.48644	−10.2202	−8.05921	−9.97953	−6.67863	−7.54998
	Best	−10.4029	−10.4027	−10.4027	−5.71945	−10.4006	−10.3165	−10.3774	−10.3792	−10.4025	−9.81922	−10.4027	−10.383	−10.0062
	Worst	−10.4029	−3.63785	−9.98411	−5.30082	−5.30082	−2.43296	−2.47727	−3.53837	−9.98285	−4.54312	−5.44475	−3.25513	−3.17664
	Std	3.42E-15	1.444153	0.161004	0.161004	1.961534	3.117332	2.608368	2.356317	0.161051	1.460086	1.054647	3.033817	1.711875
	Median	−10.4029	−10.2047	−10.296	−5.61271	−9.10019	−7.78563	−9.98165	−10.0327	−10.2957	−8.36284	−10.2334	−5.41806	−7.93751
	Rank	1	5	2	13	8	11	7	6	3	9	4	12	10
F20	Mean	−10.5364	−10.4274	−10.4274	−5.66249	−9.20887	−7.67717	−8.70667	−9.48063	−10.427	−8.26849	−10.208	−6.80118	−6.74773
	Best	−10.5364	−10.5295	−10.5295	−5.76459	−10.4527	−10.4346	−10.5286	−10.5295	−10.5293	−9.76719	−10.5295	−10.5216	−9.80024
	Worst	−10.5364	−10.1103	−10.1103	−5.34538	−5.34538	−3.35452	−2.60974	−5.38693	−10.11	−4.87011	−6.04545	−3.2291	−3.28855
	Std	2.7E-15	0.113406	0.113406	0.113407	1.381663	2.948184	2.843599	1.92362	0.113391	1.422554	0.963479	3.310305	2.211936
	Median	−10.5364	−10.4585	−10.4585	−5.69352	−9.5868	−10.0178	−10.413	−10.4331	−10.4582	−8.77756	−10.4585	−4.53964	−7.24575
	Rank	1	2	3	13	7	10	8	6	4	9	5	11	12
F21	Mean	0.397887	0.397928	0.397928	0.409123	0.398381	0.39796	0.397928	0.397928	0.397929	0.397992	0.397928	0.703449	0.457962
	Best	0.397887	0.397887	0.397887	0.39867	0.397887	0.397893	0.397887	0.397887	0.397888	0.397899	0.397887	0.397887	0.397887
	Worst	0.397887	0.398145	0.398145	0.474877	0.401023	0.398168	0.398146	0.398145	0.398145	0.398164	0.398145	2.506628	1.591156
	Std	0	7.35E-05	7.36E-05	0.016725	0.000896	8.73E-05	7.35E-05	7.36E-05	7.35E-05	8.56E-05	7.36E-05	0.61029	0.260471
	Median	0.397887	0.397894	0.397894	0.403092	0.397971	0.397918	0.397895	0.397894	0.397894	0.397973	0.397894	0.397918	0.397966
	Rank	1	4	2	10	9	7	5	3	6	8	2	12	11
F22	Mean	3	3.2491	3.249101	5.693971	6.034843	10.74003	3.249123	3.249101	3.249112	3.249101	3.2491	3.2491	7.040393
	Best	3	3.001098	3.001098	3.002107	3.013375	3.001104	3.001098	3.001098	3.001101	3.001099	3.001098	3.001098	3.003001
	Worst	3	5.127366	5.127366	27.95563	28.91822	81.45687	5.127367	5.127367	5.127377	5.127368	5.127366	5.127366	31.20499
	Std	1.14E-15	0.489279	0.489279	7.321184	5.961004	22.46757	0.489272	0.489279	0.489277	0.489279	0.489279	0.489279	8.990184
	Median	3	3.04441	3.04441	3.14002	3.541047	3.140014	3.044417	3.04441	3.04443	3.044411	3.04441	3.04441	3.179816
	Rank	1	2	6	10	11	13	9	5	8	7	4	3	12
F23	Mean	−3.86278	−3.85185	−3.85185	−3.82907	−3.7303	−3.8515	−3.84977	−3.85185	−3.85051	−3.85088	−3.85185	−3.85185	−3.85171
	Best	−3.86278	−3.86278	−3.86278	−3.85382	−3.86278	−3.86268	−3.86277	−3.86278	−3.86278	−3.86253	−3.86278	−3.86278	−3.86276
	Worst	−3.86278	−3.81789	−3.81789	−3.77776	−3.31594	−3.81781	−3.8175	−3.81789	−3.81778	−3.81766	−3.81789	−3.81789	−3.81759
	Std	2.22E-15	0.010564	0.010564	0.021113	0.12881	0.010411	0.010321	0.010564	0.010614	0.01013	0.010564	0.010564	0.010686
	Median	−3.86278	−3.85184	−3.85184	−3.83257	−3.73109	−3.8518	−3.85033	−3.85184	−3.85132	−3.85144	−3.85184	−3.85184	−3.85177
	Rank	1	2	4	11	12	7	10	5	9	8	3	3	6
Sum rank		10	44	34	106	88	102	67	51	67	74	48	81	96
Mean rank		1	4.4	3.4	10.6	8.8	10.2	6.7	5.1	6.7	7.4	4.8	8.1	9.6
Total ranking		1	3	2	12	9	11	6	5	6	7	4	8	10

**Table 4:** Optimization results of CEC 2017 test suite.

		hPSO-TLBO	WSO	AVOA	RSA	MPA	TSA	GWO	hPT2	hPT1	ITLBO	IPSO	TLBO	PSO
C17-F1	Mean	100	5.29E+09	3748.368	9.6E+09	33,159,361	1.64E+09	82,897,341	42,368,208	50,218,729	46,455,430	2.19E+09	1.38E+08	3091.392
	Best	100	4.38E+09	508.7437	8.29E+09	10,544.92	3.5E+08	26,138.81	10,715,873	14,220,698	14,179,262	1.92E+09	61,616,141	341.377
	Worst	100	6.79E+09	11272.16	1.14E+10	1.2E+08	3.56E+09	3.01E+08	78,853,365	82,349,955	75,758,100	2.6E+09	3.34E+08	9150.309
	Std	0	1.1E+09	5382.989	1.49E+09	61,500,184	1.51E+09	1.54E+08	32,783,474	35,674,006	31,933,461	3.27E+08	1.38E+08	4290.734
	Median	100	5E+09	1606.281	9.33E+09	6,078,119	1.31E+09	15,193,524	39,951,797	52,152,132	47,942,180	2.12E+09	79,005,680	1436.94
	Rank	1	12	3	13	4	10	8	5	7	6	11	9	2
C17-F3	Mean	300	8033.049	301.7791	9082.776	1340.568	10543.27	2901.42	958.6736	981.4005	863.5039	2647.886	700.4938	300
	Best	300	4074.832	300	4905.748	761.6017	4026.168	1454.004	589.3344	598.4639	546.0747	2060.398	460.8804	300
	Worst	300	10,742.63	303.8055	12,146.04	2399.799	14,898.69	5549.497	1575.43	1603.381	1365.497	3170.95	857.0187	300
	Std	0	3069.111	2.171373	3482.46	795.0646	4856.717	1987.661	471.1406	478.0596	388.2045	532.1373	182.6734	0
	Median	300	8657.364	301.6555	9639.658	1100.436	11624.12	2301.09	834.9652	861.8787	771.2216	2680.099	742.0381	300
	Rank	1	11	3	12	8	13	10	6	7	5	9	4	2
C17-F4	Mean	400	902.4571	405.3373	1295.052	407.1945	566.7586	411.9069	406.1412	407.7145	405.3359	611.6262	409.4929	419.97
	Best	400	677.5934	401.6131	818.3657	402.3049	473.6418	405.7307	402.8584	403.3799	403.6201	498.7167	407.9005	400.1039
	Worst	400	1104.07	409.1497	1760.71	411.9996	674.1198	426.6833	410.3496	414.603	407.151	713.2278	411.7909	469.1877
	Std	0	204.0081	3.31691	422.7467	5.124688	102.6733	10.46654	3.59682	5.216529	1.981138	93.99366	1.725242	34.87123
	Median	400	914.0825	405.2932	1300.566	407.2368	559.6364	407.6068	405.6784	406.4375	405.2862	617.2802	409.1402	405.2941
	Rank	1	12	3	13	5	10	8	4	6	2	11	7	9
C17-F5	Mean	501.2464	561.909	543.0492	570.3636	513.4648	562.3384	513.5991	514.2683	517.4738	517.9574	529.0633	533.5634	527.7224
	Best	500.9951	547.5217	526.9607	555.91	508.5995	543.3011	508.5861	510.1615	512.195	514.5008	519.2719	527.6244	511.0772
	Worst	501.9917	570.3438	561.9262	585.6405	518.5725	592.2042	520.7717	517.8512	522.4134	521.9358	541.1436	537.1694	551.4064
	Std	0.522698	11.04339	19.14307	17.23184	5.815881	23.14391	5.492417	4.336286	5.549772	3.400253	11.51121	4.627727	19.5741
	Median	500.9993	564.8853	541.655	569.9518	513.3437	556.9241	512.5192	514.5302	517.6433	517.6965	527.9188	534.7298	524.2029
	Rank	1	11	10	13	2	12	3	4	5	6	8	9	7
C17-F6	Mean	600	631.2476	616.8356	639.1331	601.4607	623.9964	601.397	602.1618	602.9351	603.8761	611.6091	606.8656	607.4064
	Best	600	627.3127	615.7521	636.0102	600.8105	614.5053	600.8308	601.3224	601.7229	603.1373	609.0681	604.6699	601.3504
	Worst	600	634.131	619.2083	642.9987	603.1224	638.5972	601.9612	603.9859	605.5767	604.9986	616.0634	610.5068	619.1985
	Std	0	3.305217	1.686946	3.432936	1.171408	10.83277	0.549504	1.316626	1.912893	0.905984	3.231394	2.819375	8.516125
	Median	600	631.7734	616.1909	638.7618	600.955	621.4415	601.398	601.6695	602.2204	603.6842	610.6524	606.1428	604.5383
	Rank	1	12	10	13	3	11	2	4	5	6	9	7	8
C17-F7	Mean	711.1267	799.8043	763.968	800.9678	724.9498	823.9647	726.2615	726.252	729.6364	731.7253	742.712	751.0914	732.6781
	Best	710.6726	780.3107	743.0682	788.1026	720.9932	785.4855	718.1642	723.984	727.3951	729.0775	738.5639	746.5242	725.5384
	Worst	711.7995	816.1562	790.5001	813.545	728.9161	864.0148	742.7212	728.6154	733.1142	734.773	749.4638	759.339	744.2048
	Std	0.538751	15.89892	23.05212	12.5471	3.619511	35.90389	11.9049	2.287687	2.824638	2.697238	5.23572	6.055654	8.957916
	Median	711.0174	801.3751	761.1518	801.1118	724.9449	823.1792	722.0802	726.2044	729.0182	731.5253	741.4101	749.2512	730.4845
	Rank	1	11	10	12	2	13	4	3	5	6	8	9	7
C17-F8	Mean	801.4928	847.368	830.689	852.2232	813.0826	847.0627	816.1175	814.5802	817.6934	817.3872	823.3427	836.9731	822.7208
	Best	800.995	839.9264	820.163	841.7367	809.2484	831.8296	810.8479	813.4555	816.3749	815.8313	821.7669	830.0727	815.66
	Worst	801.9912	855.1817	845.4663	857.0366	815.4123	865.287	820.5682	817.0288	820.7097	819.2567	826.6415	844.4105	829.1593
	Std	0.604721	7.476443	11.16894	7.438301	2.867818	15.79139	4.31119	1.732891	2.128168	1.740719	2.340614	7.688001	7.036328
	Median	801.4926	847.182	828.5632	855.0598	813.8348	845.5672	816.5269	813.9183	816.8444	817.2305	822.4812	836.7047	823.0318
	Rank	1	12	9	13	2	11	4	3	6	5	8	10	7
C17-F9	Mean	900	1399.012	1175.026	1441.344	905.1431	1358.747	911.5695	904.9645	905.8344	936.0052	1025.904	911.4671	904.2313
	Best	900	1262.075	951.2954	1350.309	900.3551	1155.798	900.5895	901.8209	902.4042	908.0793	1006.621	906.9387	900.897
	Worst	900	1533.658	1626.152	1572.35	912.7679	1633.582	931.6466	908.4465	908.8868	989.7544	1057.233	919.6198	912.2878
	Std	0	128.106	328.8354	99.49603	5.777535	217.3993	15.20639	2.852761	2.828695	39.22887	23.05723	5.878449	5.721816
	Median	900	1400.157	1061.328	1421.359	903.7246	1322.804	907.021	904.7952	906.0233	923.0936	1019.88	909.6549	901.8702
	Rank	1	12	10	13	4	11	7	3	5	8	9	6	2
C17-F10	Mean	1006.179	2272.674	1775.365	2531.984	1528.608	2016.237	1725.841	1517.585	1630.457	1557.721	1809.69	2147.981	1934.218
	Best	1000.284	2023.195	1480.899	2368.324	1393.692	1773.52	1533.16	1368.113	1438.83	1389.851	1638.212	1762.198	1553.546
	Worst	1012.668	2447.536	2374.322	2873.26	1616.944	2238.049	1995.308	1624.587	1775.287	1643.554	1931.07	2437.728	2335.345
	Std	7.002135	197.3598	436.2218	244.329	107.0706	266.5065	205.4292	117.2316	151.6732	120.6617	148.1346	301.1232	337.7875
	Median	1005.882	2309.983	1623.12	2443.176	1551.898	2026.69	1687.449	1538.821	1653.855	1598.741	1834.739	2195.999	1923.991
	Rank	1	12	7	13	3	10	6	2	5	4	8	11	9
C17-F11	Mean	1100	3706.841	1147.646	3823.904	1127.395	5216.321	1154.034	1124.996	1130.054	1127.69	1740.549	1149.919	1142.951
	Best	1100	2532.404	1118.884	1439.991	1114.214	5075.943	1122.162	1116.939	1121.208	1120.79	1195.462	1137.25	1131.823
	Worst	1100	4840.894	1197.819	6177.695	1158.269	5292.963	1223.95	1142.362	1148.311	1136.876	2267.18	1169.998	1164.161
	Std	0	1091.707	36.77475	2240.039	22.00052	101.5156	50.03127	12.30356	13.03865	8.484187	511.199	14.76569	15.3188
	Median	1100	3727.033	1136.941	3838.966	1118.549	5248.19	1135.013	1120.341	1125.348	1126.546	1749.777	1146.215	1137.909
	Rank	1	11	7	12	3	13	9	2	5	4	10	8	6
C17-F12	Mean	1352.959	3.34E+08	1,041,840	6.67E+08	537,442.3	984,200.6	1,339,676	1,119,517	1,391,129	1,447,652	1.52E+08	4,781,626	8018.164
	Best	1318.646	74,974,042	337,122.5	1.48E+08	19,273.83	510,668.3	43,473.74	545,184.3	617,787.2	614,496.8	33,669,030	1,279,648	2505.361
	Worst	1438.176	5.84E+08	1,889,187	1.17E+09	841,127.5	120,7984	2,097,033	1,919,043	2,399,960	2,461,224	2.65E+08	8,464,893	13,785.05
	Std	60.27339	2.71E+08	763,715.2	5.42E+08	380,785.4	345,933.8	952,033.6	661,001.8	879,737.4	953,977.1	1.23E+08	4,003,531	5405.839
	Median	1327.506	3.39E+08	970,525.5	6.77E+08	644,684	1,109,075	1,609,099	1,006,920	1,273,385	1,357,444	1.54E+08	4,690,982	7891.125
	Rank	1	12	5	13	3	4	7	6	8	9	11	10	2
C17-F13	Mean	1305.324	16,270,434	17,645.32	32530469	5441.239	12351.58	10,044.19	6291.857	7405.297	8219.529	7,388,048	16,125.5	6564.175
	Best	1303.114	1,357,783	2693.02	2700954	3735.496	7913.531	6372.143	5115.949	6074.468	6835.592	615,922	15,108.72	2367.428
	Worst	1308.508	54,003,827	29,936.8	1.08E+08	6879.06	19310	13,730	7769.705	9547.435	10,480.91	24,516,110	18,714.84	16,549.6
	Std	2.390774	26,518,937	14,969.87	53036127	1535.233	5258.8	3196.376	1212.391	1609.972	1827.786	12,037,713	1826.012	7080.253
	Median	1304.837	4,860,063	18,975.73	9,713,262	5575.2	11,091.4	10,037.31	6140.887	6999.642	7780.809	2,210,080	15,339.21	3669.834
	Rank	1	12	10	13	2	8	7	3	5	6	11	9	4
C17-F14	Mean	1400.746	3925.288	2057.876	5207.557	1980.469	3350.637	2365.338	1807.086	1903.042	1740.355	2937.621	1649.611	2980.369
	Best	1400	3067.479	1697.619	4645.47	1434.591	1489.137	1470.095	1453.804	1467.202	1498.386	2195.607	1515.859	1432.215
	Worst	1400.995	5224.087	2758.654	6608.887	2857.453	5364.252	4808.816	2285.551	2338.789	2124.347	4045.413	1833.038	6791.638
	Std	0.523309	1056.743	519.5787	986.0415	713.9192	2220.777	1716.878	441.5361	528.3317	288.3858	834.9858	140.3634	2694.358
	Median	1400.995	3704.793	1887.614	4787.935	1814.917	3274.581	1591.22	1744.495	1903.088	1669.343	2754.732	1624.773	1848.812
	Rank	1	12	7	13	6	11	8	4	5	3	9	2	10
C17-F15	Mean	1500.331	10,141.39	5420.887	13544.47	4168.477	7035.452	5909.892	3267.633	3681.831	3018.033	7377.044	2021.457	8924.747
	Best	1500.001	3199.972	2428.092	2943.897	3518.151	2551.255	3846.725	2857.078	2946.817	2337.027	4624.808	1842.257	2858.362
	Worst	1500.5	17,211.28	12098.81	28,895.1	5286.274	12348.58	6956.217	4051.452	4770.569	3748.602	9747.737	2152.946	14665.88
	Std	0.247648	6166.653	4733.27	11904.67	828.5131	4366.529	1487.545	574.9909	815.2069	631.9577	2486.011	136.7122	5191.053
	Median	1500.413	10077.16	3578.324	11169.44	3934.741	6620.985	6418.313	3081.001	3504.969	2993.251	7567.815	2045.312	9087.371
	Rank	1	12	7	13	6	9	8	4	5	3	10	2	11
C17-F16	Mean	1600.76	2004.613	1812.435	2008.33	1693.73	2037.573	1735.653	1675.247	1696.691	1675.458	1821.143	1686.788	1920.464
	Best	1600.356	1942.809	1650.286	1817.989	1654.67	1863.642	1630.12	1653.19	1675.794	1668.794	1761.02	1660.138	1820.271
	Worst	1601.12	2147.237	1924.089	2263.297	1719.019	2207.992	1823.788	1695.833	1716.189	1682.292	1862.547	1734.499	2078.647
	Std	0.332314	100.9265	121.8302	196.52	31.24997	162.9157	83.92401	20.85885	23.36918	6.773129	47.08525	34.94773	125.8979
	Median	1600.781	1964.204	1837.683	1976.017	1700.616	2039.328	1744.351	1675.982	1697.39	1675.374	1830.503	1676.257	1891.469
	Rank	1	11	8	12	5	13	7	2	6	3	9	4	10
C17-F17	Mean	1700.099	1814.266	1750.562	1814.408	1736.121	1799.078	1767.188	1731.472	1737.416	1733.435	1753.892	1757.577	1751.874
	Best	1700.02	1805.452	1734.572	1798.473	1723.307	1784.404	1725.151	1722.744	1728.511	1725.121	1749.079	1748.26	1745.29
	Worst	1700.332	1819.514	1792.558	1823.434	1773.326	1809.306	1864.923	1753.753	1760.192	1750.076	1763.547	1766.912	1758.499
	Std	0.163219	6.467607	29.52981	11.56531	26.09862	11.31541	68.90608	15.67995	16.06212	11.86723	7.104734	9.869326	5.942316
	Median	1700.022	1816.048	1737.558	1817.862	1723.925	1801.301	1739.339	1724.696	1730.481	1729.272	1751.471	1757.569	1751.853
	Rank	1	12	6	13	4	11	10	2	5	3	8	9	7
C17-F18	Mean	1805.36	2,700,242	12,164.85	5,383,877	11,402.61	12,356.16	19,768.51	12,292.58	14,853.55	12,788.06	1,232,709	28,836.63	21,629.9
	Best	1800.003	138,517.9	4723.003	266,622.3	4264.898	7199.645	6310.461	6928.172	8412.253	8741.04	66,373.95	23,000.7	2867.661
	Worst	1820.451	7,824,444	16,330.68	15,627,891	17,201.99	15,723.79	31,879.1	16,355.13	19,819.87	15,990.6	3,564,332	36,637.14	40,262.56
	Std	10.58584	3,744,655	5510.427	7,486,898	5844.873	3889.499	13,653.75	4139.17	5194.742	3184.802	1,705,209	6740.655	20,306.86
	Median	1800.492	1,419,003	13,802.86	2,820,497	12,071.78	13,250.6	20,442.24	12,943.52	15,591.04	13,210.29	650,064.1	27,854.34	21,694.7
	Rank	1	12	3	13	2	5	8	4	7	6	11	10	9
C17-F19	Mean	1900.445	375,744.6	7433.981	666,091.6	6385.277	119,677.3	6182.604	5568.259	6953.678	4601.691	16,1041.9	5533.399	24,663.69
	Best	1900.039	23,791.01	2314.747	43,418.54	2448.01	2024.057	2198.753	2410.486	2462.793	3059.651	11,808.98	2168.611	2615.743
	Worst	1901.559	791,564.4	13,172.51	1,428,796	12,225.8	240,192.4	13,706.12	9972.915	14,000.35	5757.046	32,7034.9	12,057.43	75,964.03
	Std	0.783273	347,977.3	4854.412	656805.8	4951.454	142,642.6	5427.918	3428.423	5249.274	1234.592	145,239.8	4793.22	36,380.59
	Median	1900.09	343,811.5	7124.333	596075.7	5433.649	118,246.3	4412.77	4944.818	5675.786	4795.034	152,661.8	3953.779	10,037.49
	Rank	1	12	8	13	6	10	5	4	7	2	11	3	9
C17-F20	Mean	2000.312	2210.424	2168.398	2217.963	2094.49	2203.182	2167.789	2070.211	2083.096	2073.4	2131.918	2075.291	2166.904
	Best	2000.312	2157.425	2035.901	2161.66	2077.367	2108.709	2132.315	2059.651	2073.652	2068.536	2110.495	2066.244	2143.076
	Worst	2000.312	2278.136	2286.855	2269.353	2122.343	2311.801	2238.703	2085.805	2097.633	2076.984	2146.375	2084.098	2198.36
	Std	0	52.6401	118.8435	55.96682	20.40431	90.57678	50.6873	11.6748	10.75655	4.049637	17.75158	7.780774	28.90349
	Median	2000.312	2203.068	2175.419	2220.419	2089.124	2196.11	2150.068	2067.693	2080.549	2074.04	2135.401	2075.411	2163.091
	Rank	1	12	10	13	6	11	9	2	5	3	7	4	8
C17-F21	Mean	2200	2293.113	2218.166	2268.57	2259.186	2323.447	2312.219	2253.103	2264.824	2247.422	2271.055	2299.357	2317.423
	Best	2200	2248.137	2209.252	2228.085	2256.486	2224.969	2307.894	2238.417	2245.395	2229.816	2264.436	2208.954	2309.456
	Worst	2200	2317.485	2242.32	2291.427	2261.821	2368.166	2317.154	2258.256	2271.644	2253.95	2276.721	2335.586	2324.888
	Std	0	34.11594	16.97517	29.54726	2.42295	70.30435	4.009014	10.30393	13.62872	12.38228	5.441208	63.82541	7.984688
	Median	2200	2303.416	2210.546	2277.384	2259.219	2350.326	2311.915	2257.869	2271.129	2252.961	2271.532	2326.444	2317.675
	Rank	1	9	2	7	5	13	11	4	6	3	8	10	12
C17-F22	Mean	2300.073	2713.841	2309.071	2883.186	2305.307	2692.102	2308.709	2306.569	2308.325	2307.335	2438.143	2319.089	2313.126
	Best	2300	2594.706	2304.299	2685.119	2300.92	2441.121	2301.226	2302.865	2303.674	2304.701	2389.617	2312.725	2300.631
	Worst	2300.29	2845.002	2311.883	3027.823	2309.028	2888.349	2321.372	2310.282	2312.188	2311.213	2469.466	2329.793	2344.973
	Std	0.152615	121.8081	3.496248	152.0155	3.871741	210.3358	9.356375	3.681474	4.734889	3.042804	37.56147	8.270263	22.38123
	Median	2300	2707.829	2310.05	2909.901	2305.639	2719.468	2306.119	2306.565	2308.718	2306.713	2446.746	2316.919	2303.451
	Rank	1	12	7	13	2	11	6	3	5	4	10	9	8
C17-F23	Mean	2600.919	2693.942	2641.835	2697.28	2615.498	2718.917	2614.945	2617.187	2621.763	2620.083	2640.702	2642.286	2643.937
	Best	2600.003	2654.227	2630.609	2669.522	2612.898	2634.545	2609.025	2616.18	2620.188	2618.871	2631.633	2631.733	2636.826
	Worst	2602.87	2716.599	2658.423	2735.505	2617.81	2761.529	2621.062	2618.675	2623.595	2620.645	2648.436	2650.816	2655.745
	Std	1.388886	30.84248	13.79002	32.48161	2.349835	60.1772	6.381254	1.199337	1.496877	0.865276	8.465968	8.871236	9.013826
	Median	2600.403	2702.471	2639.155	2692.047	2615.641	2739.797	2614.846	2616.947	2621.635	2620.408	2641.369	2643.297	2641.588
	Rank	1	11	8	12	3	13	2	4	6	5	7	9	10
C17-F24	Mean	2630.488	2775.69	2766.263	2844.242	2636.472	2672.139	2748.42	2658.064	2672.069	2672.271	2721.323	2755.106	2764.326
	Best	2516.677	2723.653	2734.285	2820.922	2622.02	2537.506	2724.236	2620.287	2645.719	2637.937	2695.625	2742.434	2755.438
	Worst	2732.32	2853.529	2786.472	2904.605	2643.687	2810.158	2761.841	2687.9	2692.953	2703.001	2756.65	2767.175	2785.859
	Std	122.5498	66.25804	26.01061	42.42327	10.52639	153.1914	18.113	36.73978	24.59657	36.41109	28.98253	12.15716	15.28211
	Median	2636.477	2762.789	2772.148	2825.721	2640.09	2670.446	2753.801	2662.034	2674.802	2674.073	2716.509	2755.407	2758.003
	Rank	1	12	11	13	2	5	8	3	4	6	7	9	10
C17-F25	Mean	2932.639	3147.52	2914.105	3258.12	2918.278	3122.551	2937.974	2924.361	2923.909	2924.435	2998.858	2933.081	2923.428
	Best	2898.047	3060.682	2899.066	3194.189	2915.276	2907.931	2922.626	2910.753	2911.757	2909.85	2994.502	2915.173	2898.661
	Worst	2945.793	3340.747	2948.83	3328.696	2924.512	3617.385	2945.848	2933.713	2934.162	2936.692	3003.784	2950.078	2946.546
	Std	24.28873	136.745	24.50997	58.41904	4.545926	350.9255	10.96339	10.4474	9.942083	12.25131	4.891705	20.20376	27.48149
	Median	2943.359	3094.325	2904.261	3254.798	2916.663	2982.444	2941.711	2926.489	2924.858	2925.599	2998.573	2933.536	2924.253
	Rank	7	12	1	13	2	11	9	5	4	6	10	8	3
C17-F26	Mean	2900	3564.329	2975.83	3711.483	3005.965	3583.082	3246.291	3009.708	3026.412	3022.556	3121.487	3190.688	2904.021
	Best	2900	3234.171	2811.89	3400.454	2897.241	3136.09	2970.3	2917.262	2917.91	2904.846	3091.367	2911.421	2807.879
	Worst	2900	3796.619	3140.237	4030.65	3268.876	4197.64	3850.233	3263.987	3311.448	3306.742	3165.835	3820.463	3008.206
	Std	3.91E-13	283.394	199.1308	284.9489	185.0471	548.1546	427.1786	178.4936	200.1559	200.2849	33.22064	444.7674	86.1682
	Median	2900	3613.263	2975.597	3707.413	2928.871	3499.298	3082.316	2928.793	2938.146	2939.317	3114.373	3015.434	2900
	Rank	1	11	3	13	4	12	10	5	7	6	8	9	2
C17-F27	Mean	3089.518	3204.167	3120.41	3225.692	3105.902	3176.807	3116.721	3104.304	3108.182	3104.823	3139.886	3115.756	3135.637
	Best	3089.518	3156.248	3097.214	3125.56	3092.427	3103.962	3094.506	3092.667	3093.499	3093.73	3100.949	3097.288	3097.024
	Worst	3089.518	3273.898	3180.171	3407.879	3135.533	3216.28	3176.245	3119.472	3124.883	3119.855	3180.549	3168.415	3182.511
	Std	2.76E-13	52.2364	41.98196	131.1143	21.01533	54.05908	41.81935	12.40577	14.58567	13.29019	37.02128	36.95224	37.81668
	Median	3089.518	3193.26	3102.127	3184.664	3097.825	3193.493	3098.068	3102.539	3107.174	3102.853	3139.023	3098.661	3131.506
	Rank	1	12	8	13	4	11	7	2	5	3	10	6	9
C17-F28	Mean	3100	3603.64	3237.661	3751.609	3221.039	3569.029	3340.651	3210.964	3234.096	3213.894	3350.357	3321.862	3303.482
	Best	3100	3559.755	3103.322	3668.181	3175.855	3407.301	3202.125	3198.356	3221.507	3193.392	3293.056	3215.875	3176.295
	Worst	3100	3638.239	3387.263	3810.552	3243.76	3761.461	3403.302	3222.506	3247.961	3224.092	3390.265	3387.482	3387.467
	Std	0	34.63407	131.9717	69.78185	33.67228	193.5914	97.91534	11.64192	11.86854	14.86709	44.26733	83.67492	100.7125
	Median	3100	3608.284	3230.029	3763.852	3232.269	3553.676	3378.588	3211.497	3233.458	3219.045	3359.054	3342.046	3325.084
	Rank	1	12	6	13	4	11	9	2	5	3	10	8	7
C17-F29	Mean	3132.241	3323.772	3282.346	3368.261	3205.185	3236.586	3264.015	3190.529	3202.051	3196.414	3250.557	3214.224	3264.841
	Best	3130.076	3306.05	3207.841	3296.377	3165.729	3173.711	3194.377	3165.16	3171.76	3172.48	3191.255	3171.844	3167.558
	Worst	3134.841	3340.313	3362.494	3434.075	3242.993	3298.718	3370.766	3215.251	3225.83	3225.81	3285.27	3238.474	3346.938
	Std	2.611232	18.67158	81.30356	72.1697	35.30926	53.97501	89.62088	21.55151	23.57208	24.3712	43.08385	32.07396	85.65981
	Median	3132.023	3324.362	3279.524	3371.295	3206.009	3236.958	3245.458	3190.853	3205.307	3193.684	3262.852	3223.288	3272.434
	Rank	1	12	11	13	5	7	9	2	4	3	8	6	10
C17-F30	Mean	3418.734	2,111,674	294,724.6	3,484,443	407,950.5	596,404.5	899,516.1	253,362	276,973.7	223,025.7	1,045,246	73,878.61	382,013.5
	Best	3394.682	1,277,340	99,075.07	781,071.3	15,417.93	138,930.3	32,077.15	14,510.35	16,141.98	30,921.79	354,534.7	28,022.71	6354.214
	Worst	3442.907	3,145,244	757,357.9	5,477,669	61,0461.1	122,6181	1,291,826	374,902.6	417,356.2	313,368.7	1,361,416	129,022	757,392.4
	Std	29.21253	813,970	326,040.1	2,072,605	280,538.3	484,692.4	624,972.1	170,483.8	188,716.3	136,522.3	488,851.6	43,961.79	455,349.8
	Median	3418.673	2,012,056	161,232.7	3,839,517	502,961.4	510,253.1	1,137,081	312,017.5	337,198.3	273,906.2	1,232,517	69,234.87	382,153.8
	Rank	1	12	6	13	8	9	10	4	5	3	11	2	7
Sum rank		35	338	199	366	115	299	211	101	160	132	267	209	207
Mean rank		1.206897	11.65517	6.862069	12.62069	3.965517	10.31034	7.275862	3.482759	5.517241	4.551724	9.206897	7.206897	7.137931
Total rank		1	12	6	13	3	11	9	2	5	4	10	8	7

**Table 5:** Wilcoxon rank sum test results.

Compared Algorithms	Unimodal	High-Multimodal	Fixed-Multimodal	CEC 2017 Test Suite
hPSO-TLBO vs. WSO	1.85E-24	1.97E-21	2.09E-34	2.02E-21
hPSO-TLBO vs. AVOA	3.02E-11	4.99E-05	1.44E-34	3.77E-19
hPSO-TLBO vs. RSA	4.25E-07	1.63E-11	1.44E-34	1.97E-21
hPSO-TLBO vs. MPA	1.01E-24	1.04E-14	2.09E-34	2.00E-18
hPSO-TLBO vs. TSA	1.01E-24	1.31E-20	1.44E-34	9.50E-21
hPSO-TLBO vs. GWO	1.01E-24	5.34E-16	1.44E-34	5.23E-21
hPSO-TLBO vs. hPT2	1.01E-24	1.51E-22	1.44E-34	5.88E-20
hPSO-TLBO vs. hPT1	1.01E-24	4.09E-17	1.44E-34	3.41E-22
hPSO-TLBO vs. ITLBO	1.01E-24	5.34E-16	1.44E-34	2.40E-22
hPSO-TLBO vs. IPSO	1.01E-24	2.46E-24	1.44E-34	1.04E-19
hPSO-TLBO vs. TLBO	1.01E-24	1.97E-21	1.44E-34	1.60E-18
hPSO-TLBO vs. PSO	1.01E-24	1.97E-21	1.44E-34	1.54E-19

**Table 6:** Performance of optimization algorithms on pressure vessel design problem.

Algorithm	Optimum Variables				Optimum Cost
	*T_s_*	*T_h_*	*R*	*L*	
hPSO-TLBO	0.778027	0.384579	40.31228	200	5882.901
WSO	0.778027	0.384579	40.31228	200	5882.901
AVOA	0.778031	0.384581	40.31251	199.9969	5882.909
RSA	1.266864	0.684455	64.03621	21.84755	8083.221
MPA	0.778027	0.384579	40.31228	200	5882.901
TSA	0.779753	0.386033	40.39931	200	5913.936
GWO	0.778534	0.386025	40.32206	199.9583	5891.47
hPT1	0.863331	0.551663	43.82355	178.1357	7423.859
hPT2	0.909754	0.612768	45.4607	170.1978	8203.294
ITLBO	1.007644	0.429869	44.41372	164.2482	7173.881
IPSO	0.971381	0.574936	45.31477	185.8739	8924.884
TLBO	1.697384	0.497968	48.96822	111.6649	11,655.86
PSO	1.683083	0.664227	67.07266	23.90255	10,707.79

**Table 7:** Statistical results of optimization algorithms on pressure vessel design problem.

Algorithm	Mean	Best	Worst	Std	Median	Rank
hPSO-TLBO	5882.895451	5882.895451	5882.895451	2.06E-12	5882.895451	1
WSO	5892.660121	5882.901051	5979.188336	28.7049213	5882.901464	3
AVOA	6277.54171	5882.908511	7246.78008	455.2164111	6076.08962	5
RSA	13,534.14797	8083.221035	22,422.75871	4039.895167	12,354.52124	9
MPA	5882.901057	5882.901052	5882.901064	4.76E-06	5882.901055	2
TSA	6338.024708	5913.936056	7131.963127	430.4115812	6188.536588	6
GWO	6034.674549	5891.469631	6806.784466	309.2651669	5901.245264	4
hPT1	11,215.46634	7423.857014	16,642.53656	2954.126869	11,038.72283	8
hPT2	13,923.19832	8203.292711	21,021.48088	4224.527187	13,968.72996	10
ITLBO	11,172.03452	7173.87948	18,660.95934	3548.614934	10,397.25346	7
IPSO	15,785.03122	8924.882242	22,541.58427	5160.561356	16,389.87266	11
TLBO	32,131.25646	11,655.86208	69,689.83545	17,822.77646	28,265.18798	12
PSO	33,789.17406	10,707.79023	58,436.51582	16,685.46389	37,331.59553	13

**Table 8:** Performance of optimization algorithms on speed reducer design problem.

Algorithm	Optimum Variables							Optimum Cost
	b	*M*	*p*	*l*_1_	*l*_2_	*d*_1_	*d*_2_	
hPSO-TLBO	3.5	0.7	17	7.3	7.8	3.350215	5.286683	2996.348
WSO	3.5	0.7	17	7.30001	7.8	3.350215	5.286683	2996.348
AVOA	3.5	0.7	17	7.300001	7.8	3.350215	5.286683	2996.348
RSA	3.595192	0.7	17	8.25192	8.27596	3.355842	5.489744	3188.946
MPA	3.5	0.7	17	7.3	7.8	3.350215	5.286683	2996.348
TSA	3.513321	0.7	17	7.3	8.27596	3.350551	5.290332	3014.45
GWO	3.500662	0.7	17	7.305312	7.8	3.364398	5.28888	3001.683
hPT1	3.501176	0.700321	17.46705	7.397422	7.849971	3.382102	5.297636	2.33E+10
hPT2	3.50256	0.700562	17.62741	7.461131	7.866856	3.397713	5.307414	3.86E+10
ITLBO	3.511587	0.700826	18.92588	7.46553	7.871304	3.414912	5.297564	3466.045
IPSO	3.521574	0.700022	17.33948	7.52107	7.91627	3.530869	5.345062	3161.188
TLBO	3.557936	0.704128	26.62939	8.12765	8.156521	3.673703	5.341085	5344.833
PSO	3.508452	0.700074	18.13159	7.402286	7.870261	3.603493	5.345904	3312.579

**Table 9:** Statistical results of optimization algorithms on speed reducer design problem.

Algorithm	Mean	Best	Worst	Std	Median	Rank
hPSO-TLBO	2996.348165	2996.348165	2996.348165	1.03E-12	2996.348165	1
WSO	2996.640981	2996.348305	2998.87965	0.665661946	2996.364895	3
AVOA	3001.003783	2996.348187	3011.558199	4.516285408	3000.900984	4
RSA	3285.981388	3188.946352	3346.202854	65.46309514	3301.347252	7
MPA	2996.348168	2996.348165	2996.348178	3.62E-06	2996.348166	2
TSA	3033.306292	3014.450491	3047.487651	11.54079978	3035.152884	6
GWO	3004.8929	3001.683252	3011.053403	2.85373292	3004.357996	5
hPT1	1.60763E+13	2,328,270,4326	7.95377E+13	2.14493E+13	8.15597E+12	9
hPT2	2.4969E+13	38,611,102,157	1.07078E+14	3.07571E+13	1.42599E+13	10
ITLBO	1.44E+13	3466.045209	1.04E+14	2.64E+13	5.63E+12	8
IPSO	3.18058E+13	3161.188406	1.6112E+14	4.23337E+13	2.2749E+13	11
TLBO	7.18E+13	5344.833366	5.20E+14	1.32E+14	2.81E+13	12
PSO	1.06E+14	3312.579176	5.37E+14	1.41E+14	7.58E+13	13

**Table 10:** Performance of optimization algorithms on welded beam design problem.

Algorithm	Optimum Variables				Optimum Cost
	*h*	*l*	*t*	*b*	
hPSO-TLBO	0.20573	3.470489	9.036624	0.20573	1.724852
WSO	0.20573	3.470489	9.036624	0.20573	1.724852
AVOA	0.20494	3.487615	9.036514	0.205735	1.725954
RSA	0.196401	3.53676	9.953681	0.218189	1.983572
MPA	0.20573	3.470489	9.036624	0.20573	1.724852
TSA	0.204146	3.496185	9.065083	0.20617	1.734136
GWO	0.205588	3.473748	9.036228	0.205801	1.725545
hPT1	0.237138	3.829949	8.522555	0.262167	2.139874
hPT2	0.243247	3.783904	9.178428	0.263847	2.384718
ITLBO	0.227451	3.687204	8.574407	0.25102	1.994317
IPSO	0.268698	3.523407	8.892821	0.293392	2.53362
TLBO	0.318796	4.452332	6.725274	0.432185	3.065577
PSO	0.377926	3.423201	7.289954	0.585841	4.097012

**Table 11:** Statistical results of optimization algorithms on welded beam design problem.

Algorithm	Mean	Best	Worst	Std	Median	Rank
hPSO-TLBO	1.724679823	1.724679823	1.724679823	2.51E-16	1.724679823	1
WSO	1.724844362	1.724844016	1.724849731	1.42E-06	1.724844016	3
AVOA	1.762377344	1.725945958	1.846469707	0.041484186	1.748038057	6
RSA	2.19632836	1.983563906	2.555158029	0.163966432	2.170484224	7
MPA	1.724844021	1.724844017	1.724844028	3.81E-09	1.724844021	2
TSA	1.743730267	1.734127479	1.753218931	0.006376703	1.743829634	5
GWO	1.727321573	1.725537072	1.731495767	0.001550229	1.727068458	4
hPT1	7.51754E+12	2.139816676	4.96972E+13	1.39052E+13	1.47507E+11	9
hPT2	1.16016E+13	2.384677086	6.62629E+13	1.94524E+13	2.95014E+11	10
ITLBO	6.87E+12	1.994259593	6.63E+13	1.85E+13	2.548744746	8
IPSO	1.4204E+13	2.533578588	8.59849E+13	2.98947E+13	3.397180524	11
TLBO	3.43E+13	3.065568295	3.31E+14	9.23E+13	5.819237012	12
PSO	4.73E+13	4.097004136	2.87E+14	9.96E+13	6.891186011	13

**Table 12:** Performance of optimization algorithms on tension/compression spring design problem.

Algorithm	Optimum Variables			Optimum Cost
	*d*	*D*	*P*	
hPSO-TLBO	0.051689	0.356718	11.28897	0.012665
WSO	0.051687	0.356669	11.29185	0.012665
AVOA	0.051176	0.344499	12.04499	0.01267
RSA	0.050081	0.312796	14.82157	0.013174
MPA	0.051691	0.35676	11.28651	0.012665
TSA	0.050966	0.339564	12.38189	0.012682
GWO	0.051965	0.363368	10.91381	0.012671
hPT1	0.055007	0.46737	9.513398	0.013657
hPT2	0.056665	0.522698	8.628156	0.014153
ITLBO	0.054843	0.4635	9.776336	0.013664
IPSO	0.056312	0.512716	9.339243	0.014227
TLBO	0.068247	0.908916	2.446611	0.017633
PSO	0.068162	0.905704	2.446611	0.017528

**Table 13:** Statistical results of optimization algorithms on tension/compression spring design problem.

Algorithm	Mean	Best	Worst	Std	Median	Rank
hPSO-TLBO	0.012601907	0.012601907	0.012601907	7.58E-18	0.012601907	1
WSO	0.012673576	0.012662188	0.012826009	4.02E-05	0.012662617	3
AVOA	0.013352445	0.012667288	0.014177381	0.000625752	0.013282895	7
RSA	0.013254044	0.013170803	0.013400678	7.79E-05	0.013232604	6
MPA	0.012662191	0.012662188	0.0126622	3.20E-09	0.01266219	2
TSA	0.012964934	0.012679454	0.013539129	0.000271138	0.012889919	5
GWO	0.012720992	0.012667804	0.012948444	6.20725E-05	0.012718442	4
hPT1	1.06544E+12	0.013636157	1.89059E+13	4.66181E+12	0.013724578	10
hPT2	2.13088E+12	0.014137489	3.78117E+13	9.32E+12	0.014252841	11
ITLBO	0.013814906	0.013642726	0.013994358	0.000119693	0.013805438	8
IPSO	6.39263E+12	0.014211676	1.13435E+14	2.79708E+13	0.014234198	12
TLBO	1.82E-02	0.017629673	1.88E-02	4.02E-04	0.018126014	9
PSO	2.13E+13	0.017524526	3.78E+14	9.32E+13	0.017524526	13

## Data Availability

Data are contained within the article.
